# TRIM25 promotes glioblastoma progression by stabilizing HIF-1α expression in normoxia through K11/K29 polyubiquitination

**DOI:** 10.1038/s41419-026-08757-3

**Published:** 2026-04-22

**Authors:** Hui Huang, Kaixiang Ni, Chenhua Li, Maorong Cai, Yuankun Liu, Jiahao Zhang, Yifan Shen, Yuning Chen, Jun Sun, Junfei Shao, Yi Liu, Wei Ji, Jiantong Jiao

**Affiliations:** 1 Department of Neurosurgery, The Affiliated Wuxi People’s Hospital of Nanjing Medical University, Wuxi, Jiangsu China; 2 Ottawa Hospital Research Institute, Regenerative Medicine Program, Ottawa, ON Canada; 3 Department of Cellular and Molecular Medicine, Faculty of Medicine, University of Ottawa, Ottawa, ON Canada

**Keywords:** CNS cancer, Ubiquitylation

## Abstract

Glioblastoma (GBM) frequently activates hypoxia signaling even under normoxic conditions, yet the mechanism sustaining hypoxia-inducible factor-1α (HIF-1α) stability remains unclear. Here, we identify the E3 ubiquitin ligase TRIM25 as a key driver of this phenomenon. TRIM25, aberrantly upregulated in GBM, directly binds HIF-1α and catalyzes K11/K29-linked polyubiquitination at lysine 532 of hydroxylated HIF-1α, preventing its canonical proteasomal degradation. This non-canonical ubiquitin modification stabilizes HIF-1α despite normal oxygen availability and sustains a pseudohypoxic transcriptional program in GBM cells. Functional studies in GBM cell lines, patient-derived cultures, and tumor models demonstrate that TRIM25-mediated HIF-1α stabilization promotes tumor proliferation, invasion, and angiogenic potential. Importantly, small-molecule screening identified T7117 as an inhibitor that disrupts the TRIM25–HIF-1α interaction, suppresses tumor growth, and enhances temozolomide efficacy. Together, our findings uncover a previously unrecognized ubiquitin mechanism that stabilizes hydroxylated HIF-1α under normoxia, revealing the TRIM25–HIF-1α axis as a driver of GBM pseudohypoxia and a potential therapeutic target.

## Introduction

Glioblastoma (GBM) is the most common and aggressive primary tumor of the central nervous system, characterized by high mortality and recurrence rates [1, 2]. Surgical resection followed by chemoradiotherapy remains the standard first-line treatment; nevertheless, the clinical outcome of patients with GBM remains extremely unfavorable. The median overall survival is approximately 15 months, with a five-year survival rate below 5% [3, 4]. A defining feature of GBM is the presence of heterogeneous oxygen availability within tumors, where hypoxic and normoxic regions coexist [5–8]. Notably, GBM cells frequently display activation of hypoxia-associated signaling pathways even under normoxic conditions, a phenomenon referred to as pseudohypoxia, which drives tumor proliferation, migration, and invasion [9–11]. Understanding the molecular basis of this pseudohypoxic state is therefore critical for identifying new therapeutic targets [12].

Although hypoxia-driven HIF-1α activation has been extensively studied in GBM, most investigations have focused on its regulation under low-oxygen conditions [13]. In contrast, the mechanisms that sustain stable HIF-1α expression under normoxia remain poorly understood. Emerging evidence suggests that post-translational regulation, particularly ubiquitin-mediated modification, plays a central role in controlling HIF-1α stability. However, the specific E3 ubiquitin ligase responsible for maintaining HIF-1α stability in normoxic GBM cells has not been identified, representing a critical gap in our understanding of GBM pseudohypoxia.

Hypoxia-inducible factors (HIFs) are central regulators of cellular adaptation to hypoxia. These transcriptional complexes consist of an oxygen-sensitive α subunit and a constitutively expressed β subunit. Under normoxia, Prolyl hydroxylases (PHD1-3), especially PHD2, hydroxylate HIF-1α, enabling recognition by the E3 ubiquitin ligase VHL and subsequent K48-linked ubiquitination and proteasomal degradation [14]. Under hypoxia, this hydroxylation process is suppressed, allowing HIF-1α to accumulate and activate transcriptional programs that promote angiogenesis, metabolic reprogramming, and tumor progression [15, 16]. However, numerous studies have reported that HIF-1α can remain abnormally stabilized in tumors even under normoxic conditions, enabling malignant cells to maintain hypoxia-associated transcriptional programs independent of oxygen availability [12, 17, 18]. This stability may be attributed to mechanisms unrelated to hypoxia itself, such as the accumulation of tumor metabolites (e.g., succinate and fumarate), mitochondrial reactive oxygen species (ROS), or epigenetic alterations that suppress the activity of PHDs [11, 19–22]. The mechanisms sustaining HIF-1α stability in normoxic GBM remain incompletely understood. In this study, we identify the E3 ubiquitin ligase TRIM25 as a key regulator of HIF-1α stability in GBM cells under normoxic conditions. TRIM25, a member of the tripartite motif-containing protein family involved in diverse ubiquitin-dependent regulatory processes, was found to interact with HIF-1α and promote its stabilization through non-canonical ubiquitination. Mechanistically, TRIM25 mediates K11/K29-linked polyubiquitination of hydroxylated HIF-1α, thereby preventing its proteasomal degradation and sustaining pseudohypoxic signaling in normoxic GBM cells. Furthermore, pharmacological targeting of the TRIM25–HIF-1α axis enhances the anti-tumor efficacy of temozolomide (TMZ), highlighting this pathway as a potential therapeutic vulnerability in GBM.

## Methods and Materials

### Clinical samples and immunohistochemistry (IHC)

Formalin-fixed paraffin-embedded and fresh glioma specimens were obtained from patients undergoing surgical resection at the Department of Neurosurgery, Affiliated Wuxi People’s Hospital of Nanjing Medical University. A total of 60 clinically annotated glioma specimens were enrolled for IHC analyses, including 5 non‑tumorous (NT) brain tissues, 14 grade II, 20 grade III, and 21 grade IV (GBM) gliomas, all of which were histopathologically diagnosed and graded according to the WHO classification of central nervous system tumors and collected from treatment‑naive patients without prior radiotherapy or chemotherapy. In addition, fresh tumor tissues from 7 GBM patients were used to establish patient‑derived primary GBM cell cultures. All patients provided written informed consent, and all sample collection and research procedures were approved by the Institutional Review Board and Ethics Committee of the Affiliated Wuxi People’s Hospital of Nanjing Medical University (No. (2023)109). Specimens with contamination, insufficient tissue quantity, or from patients with other concurrent malignant tumors were excluded. The antibodies used for the detection of the selected proteins are listed in Supplementary Table 1. IHC was performed using a standard protocol, and tissue slides were counterstained with crystal violet. HIF-1α and TRIM25 expressions were semiquantitatively assessed using an established immunoreactivity score (IRS) system [23, 24]. All slides were independently examined and scored by two pathologists blinded to the experiment, and the mean value of the IRS was considered as the final score (Supplementary Table 2).

### Online database analysis

Gene expression profile and clinicopathological data from the Chinese Glioma Genome Atlas (CGGA), The Cancer Genome Atlas (TCGA), Rembrandt, and Gravendeel databases were collected using the GlioVis data portal (http://gliovis.bioinfo.cnio.es/). Data on E3-Ligase and their E3 substrate interactions were obtained from the UbiNet 2.0 database (https://awi.cuhk.edu.cn/~ubinet/index.php).

### RNA sequencing (RNA-seq) and data analysis

The RNA-seq data from 12 primary GBM cell lines cultured under normoxic conditions were obtained from the Gene Expression Omnibus (GEO) database (https://www.ncbi.nlm.nih.gov/geo/) (GSE15824), and differential expression analysis was performed using the DESeq2 package.

Total RNA was extracted from U251 cells of both control and shTRIM25 groups using TRIzol reagent (Takara, Cat. 9109, Tokyo, Japan) according to the manufacturer’s instructions. The isolated RNA was purified and subjected to RNA-seq on the Illumina Novaseq platform. Raw FASTQ sequencing reads were trimmed using in-house Perl scripts to generate clean data. Transcript quantification was performed using featureCounts, and gene expression was represented as fragments per kilobase of transcript per million mapped reads (FPKM). Differential expression between the two groups (with or without biological replicates) was analyzed using the DESeq2 or edgeR R package. Genes with an adjusted *P* value < 0.05 (and an absolute fold change of 2 for edgeR) were considered differentially expressed. All sequencing and bioinformatic analyses were performed by GeneChem Company (Shanghai, China). The RNA-seq data were deposited in the NCBI Sequence Read Archive (SRA) under the accession number PRJNA1347646.

### Signaling signature analysis

The HIF signaling pathway signature was obtained from the Molecular Signatures Database (MSigDB, standard name: BIOCARTA_HIF_PATHWAY, systematic name: M13324) (https://www.gsea-msigdb.org/gsea/msigdb/human/geneset/BIOCARTA_HIF_PATHWAY.html). Data on E3 Ligases were obtained from the UbiNet 2.0 database (Database of E3-Substrate Interactions, data type: categorized human E3 ligases) (https://awi.cuhk.edu.cn/~ubinet/download.php). The pathway signature score was calculated using the Gene Set Enrichment Analysis (GSEA) tool.

### Cell culture

Human GBM cell lines U251 and U118, human cardiac microvascular endothelial cells (HCMECs), and the human embryonic kidney cell line 293 T were purchased from the Cell Bank of Type Culture Collection of the Chinese Academy of Sciences (Shanghai, China). All cells were maintained in Dulbecco’s modified Eagle’s medium (DMEM) supplemented with 10% fetal bovine serum (FBS; Gibco, Carlsbad, CA, USA) and incubated at 37 °C under 5% CO_2_ and 21% O_2_. Human Astrocytes (HA) cells were purchased from Procell system (CP-H122) and cultured in HA Cell Complete Medium (Procell system, CM-H122). Cell line authentication was performed by Genewiz, Inc. (Shanghai, China) using short tandem repeat profiling, and all cell lines were confirmed as mycoplasma-free (last tested in 2021). Fresh GBM samples were collected within 30 minutes after surgical resection, washed, minced, and enzymatically dissociated to generate a single-cell suspension. The isolated tumor cells were cultured in DMEM/F12 supplemented with 10% FBS and incubated at 37 °C under 5% CO_2_. After immunostaining-based identification using Nestin and GFAP antibodies, second- or third-passage cells were used for tumor xenograft generation and western blot analysis. All cell-based experiments were performed with at least three biological replicates (independent cell cultures) and two technical replicates per biological replicate, unless otherwise stated. Biological replicates refer to independent cell culture batches, and technical replicates refer to parallel detection of the same sample to ensure the reliability of the results. Cells were excluded from analysis if they showed signs of contamination or abnormal morphology during culture.

### Vector construction and transduction

The His-tagged TRIM25 lentiviral construct was generated by inserting the corresponding cDNA sequence into the GV712 lentiviral vector (Genechem, Shanghai, China). The RING domain plasmid and TRIM25 deletion mutant were derived from the wild-type TRIM25 plasmid by site-directed cloning. HIF-1α expression plasmids were constructed by inserting the cDNA sequence into the GV712 vector containing either a Flag or an HA tag. Lentiviral short hairpin RNA (shRNA) targeting human TRIM25 was purchased from GeneChem (Shanghai, China). The target cDNA sequences were as follows: 1. 5′-GTGCCCGATTCCTCTTAGAGA-3′, 2. 5′-GAACTGAACCACAAGCTGATA-3′, 3. 5′-CCAGCTCACATCCGAACTCAA-3′. A scrambled shRNA was used as a negative control (NC), and the target sequence was cloned into the GV493 lentiviral vector (GeneChem). For ubiquitination assay validation, two complementary ubiquitin mutant sets were used: (1) Single lysine-to-arginine (KxR) mutants, in which individual lysine residues were mutated to block specific ubiquitin chain formation; (2) Single lysine-only (KxO) mutants, in which all lysine residues except the indicated one were mutated to allow only the assembly of the target ubiquitin chain. All ubiquitination assays included appropriate controls to ensure specificity and reproducibility: wild-type ubiquitin as the positive control, empty vector as the negative control, and input controls to verify equal protein loading. Transfection was performed using the X-tremeGENE siRNA transfection reagent (Roche, Cat. 4476093001, Mannheim, Germany).

### Double immunofluorescence (IF) staining

Double IF staining was performed according to our previously established protocol [25]. The antibodies used to detect the selected proteins are listed in Supplementary Table 1.

### Western blot

A standard Western blot protocol was used to measure protein expression. The antibodies used to detect the selected proteins are listed in Supplementary Table 1.

### Immunoprecipitation (IP) assay

IP was performed according to our previously established protocol [25]. The antibodies used to perform IP are listed in Supplementary Table 1.

### Reverse-transcription quantitative PCR (RT-qPCR)

RT-qPCR was performed according to our previously established protocol [25, 26]. GAPDH was used as the internal control. Primer sequences are listed in Supplementary Table 3. Relative gene expression was calculated using the 2^-ΔΔCT^ method.

### Enzyme-linked immunosorbent assay (ELISA)

VEGFA concentration in the 293 T cells was measured using an ELISA kit (ab119566, Abcam) according to the manufacturer’s instructions. The optical density (OD) value was measured at 450 nm using a microplate reader.

### Luciferase assay

The reporter construct pGL4.47 [Luc2P/HRE/Hygro] was used to evaluate HIF-1α transcriptional activity. The construct contained five copies of the hypoxia-responsive element (HRE) that drove the luciferase reporter gene *luc2P* expression. The pRL-TKRenilla luciferase plasmid transfection and detailed procedures were performed according to our previously established protocol [25].

### Glutathione S-transferase (GST) pull-down assay

GST assay was performed according to our previously established protocol.

### In vitro invasion assay

Cell invasion assay was performed according to our previously established protocol [26]. Each experiment was repeated at least three times.

### Cell growth and colony formation assay

Cell growth and colony formation assay were performed according to our previously established protocol [24]. Each experiment was repeated at least six times.

### 3D-Spheroid formation and growth measure

Tumor sphere formation was assessed as previously described, with minor modifications. Images of tumor spheres were taken at specific time points, and their area was measured using ImageJ. Sphere growth was expressed as the ratio of the mean area at each time point to that measured after 1 day of culture.

### Tube formation assay

A Matrigel solution (Corning, Cat. 356234, New York, USA) was added into the wells of a prechilled 48-well sterile plate, which was incubated at 37 °C for 30 min to allow gel polymerization. HCMECs (4 × 10^4^ cells) were then seeded onto the solidified Matrigel in each well and incubated for 12 h. Subsequently, 50 µL calcein (20 nM, YEASEN, Cat. 40728ES03, China) was added to each well and cells were incubated at room temperature for 30 min in the dark. Tube formation was observed and imaged using an EVOS microscope, and total tube length was quantified using ImageJ software. The results were expressed as the mean length from 5 randomly selected tubes.

### Animal experiments

Four-week-old female BALB/c nude mice (12–16 g) were purchased from the Shanghai Animal Center, Chinese Academy of Sciences, and housed under specific pathogen-free (SPF) conditions at the Affiliated Wuxi People’s Hospital of Nanjing Medical University (temperature 20–26 °C, humidity 30–70%, 12 h light/dark cycle, maximum 5 mice per cage). After 7 days of acclimatization, mice were stratified by body weight and randomly assigned to groups using a computer-generated random number table (*n* = 6 per group for bioluminescent imaging and tissue collection; *n* = 8 per group for survival analysis). On day 0, 5 × 10⁵ glioblastoma cells were implanted into the right cerebral cortex by an investigator blinded to group allocation. Beginning 3 days after tumor implantation, mice received intraperitoneal injections of 10 μg T7117, TMZ, or vehicle every 2 days. Drug preparation and coding were performed by an independent researcher to maintain allocation concealment. All outcome assessments, including general health, body weight measurement every 5 days, and bioluminescent imaging, were conducted in a single-blinded manner. Humane endpoints were applied in accordance with ARRIVE guidelines, and mice were euthanized via cervical dislocation once meeting euthanasia criteria. Pre-established exclusion criteria were applied to the tissue collection group, while no mice were excluded from survival analysis. After exclusion, 3–4 eligible mice per group were used for tissue harvest; brains, livers, and lungs were collected, fixed in 4% formaldehyde, cryoprotected in 30% sucrose, and cryosectioned for H&E staining. All animal procedures were approved by the Ethics Committee of the Affiliated Wuxi People’s Hospital of Nanjing Medical University (No. (2023)114) and performed in compliance with ARRIVE guidelines and institutional animal care regulations.

### T7117 synthesis and molecular docking analysis

The crystal structure of the human TRIM25 protein (PDB ID: 5FER) was downloaded from the Protein Data Bank (PDB) database. Protein preparation was performed using UCSF Chimera by assigning AMBER14SB atomic charges, while protonation states at neutral pH were determined using the H++3 web server. Potential binding sites were predicted using SiteMap. The small-molecule library of FDA-approved drugs was downloaded from DrugBank through the ZINC database, and compound structures were batch-converted to PDBQT format using the Raccoon software. Molecular docking was performed using AutoDock Vina 1.2.0 software. Potential candidate compounds targeting TRIM25 were selected according to the docking score. Three-dimensional visualization of docking poses was performed using the open-source software PyMOL 2.04, and 2D interaction images were generated using the academic version of the software Maestro 12.08.

### Statistical analysis

Each experiment was repeated three times to ensure reproducibility. Data normality was verified using the Shapiro-Wilk test, and homogeneity of variance was confirmed by Levene’s test prior to parametric statistical analyses. All statistical analyses were performed using GraphPad Prism 8 software. Two-tailed Student’s t-test was used to compare two groups, while one-way ANOVA followed by Tukey’s post-hoc test (for multiple comparisons correction) was used to compare multiple groups. Survival curves were analyzed by the Kaplan-Meier method and the log-rank test. The association between gene expressions was evaluated by Spearman correlation. Results were expressed as mean ± SD of at least three independent experiments. A value of *p* < 0.05 was considered statistically significant. The sample size was determined based on the effect size (Cohen’s d = 0.7) estimated from pilot experiments, using G*Power 3.1 software with a significance level (α) of 0.05 and statistical power of 0.8.

## Results

### GBM cells exhibit pseudohypoxic signaling under normoxic conditions

**Fig. 1:**
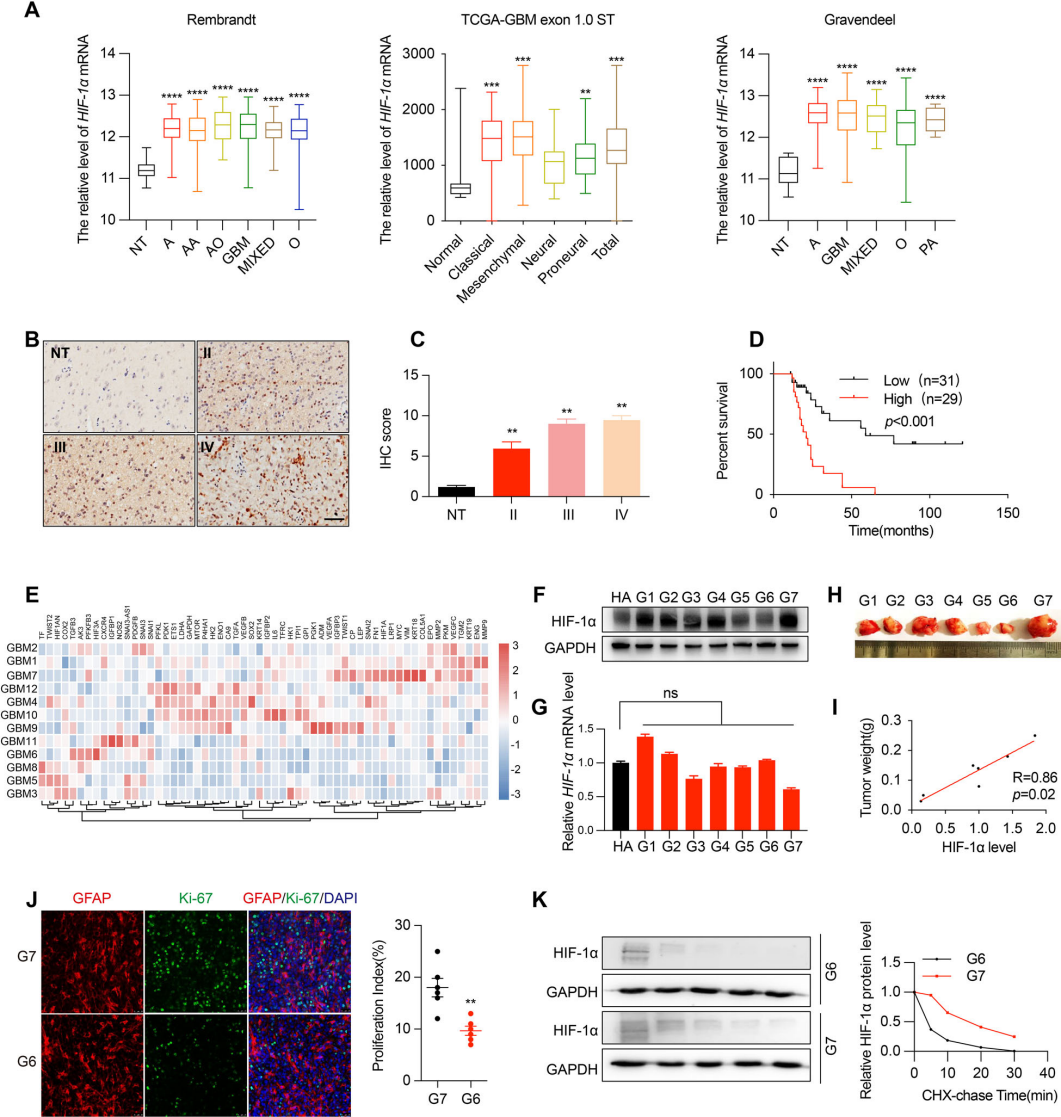
GBM cells exhibit pseudohypoxic signaling under normoxic conditions. **A** Rembrandt, TCGA, and Gravendeel databases analyzed HIF-1α expression in different glioma types. **B** Schematic representation of HIF-1α IHC in different grades of glioma tissue microarrays (Scale bar, 20 μm). **C** HIF-1α expression in different grades of gliomas (NT: 5 cases; grade II: 14 cases; grade III: 20 cases; grade IV: 21 cases). **D** Kaplan–Meier analysis of HIF-1α (cut-off: mean value of tumor expression). **E** Heatmap of the expression of key molecules and target genes of the HIF-1 signaling pathway in GBM primary cell lines under normoxia in the GEO database (GSE15824). **F** Immunoblot showing HIF-1α protein expression in HA and 7 GBM primary cell lines under normoxia. **G** RT-qPCR showing *HIF-1α* mRNA expression in HA and 7 GBM primary cell lines under normoxia. **H** Subcutaneous tumorigenicity of 7 GBM primary cell lines in nude mice. **I** Statistical analysis of the correlation between HIF-1α expression and tumorigenicity in GBM primary cell lines. **J** Cellular IF showing Ki-67 expression in tumors formed by GBM primary cell lines (×20-fold). **K** Immunoblot showing HIF-1α protein expression in GBM primary cell line G6 and G7 after protein half-life assay. NT: normal brain tissue; HA: human normal astrocytes, *p < 0.05, **p < 0.01, ***p < 0.001.

Database analysis revealed that HIF-1α mRNA expression was significantly elevated in GBM than in normal brain tissues (Fig. 1A). Consistently, immunohistochemistry (IHC) showed a marked increase in HIF-1α protein levels in grade II to IV gliomas relative to normal tissues (Fig. 1B, C) and high HIF-1α expression was associated with poor overall survival regardless of tumor grades (Fig. 1D). While HIF-1α regulation in GBM under hypoxia has been extensively studied, the mechanisms that sustain HIF-1α expression after removal from hypoxic environments remain unclear. RNA-seq analysis of primary GBM cells cultured under normoxic conditions (GSE15824) showed persistent expression of multiple HIF-1 target genes, suggesting that GBM cells retained hypoxia-like transcriptional programs even in the presence of normal oxygen levels (Fig. 1E). Consistently, HIF-1α protein levels, but not mRNA expression, was significantly higher in GBM cells compared with normal astrocytes (Fig. 1F, G), suggesting that HIF-1α accumulation may be regulated at the post-transcriptional level. Primary GBM cells with high HIF-1α expression displayed greater tumorigenicity in nude mice (Fig. 1H, I), and tumor sections revealed a positive correlation between HIF-1α and Ki-67 expression, reflecting enhanced tumor proliferation (Fig. 1J). Moreover, cycloheximide (CHX) chase assays demonstrated prolonged HIF-1α half-life in GBM cells, indicating increased protein stability (Fig. 1K). Collectively, these results indicate that GBM cells maintain a pseudohypoxic phenotype under normoxia through impaired degradation of HIF-1α, which may contribute to tumor aggressiveness.

### TRIM25 activates the HIF-1 signaling pathway by stabilizing HIF-1α expression under normoxia

**Fig. 2:**
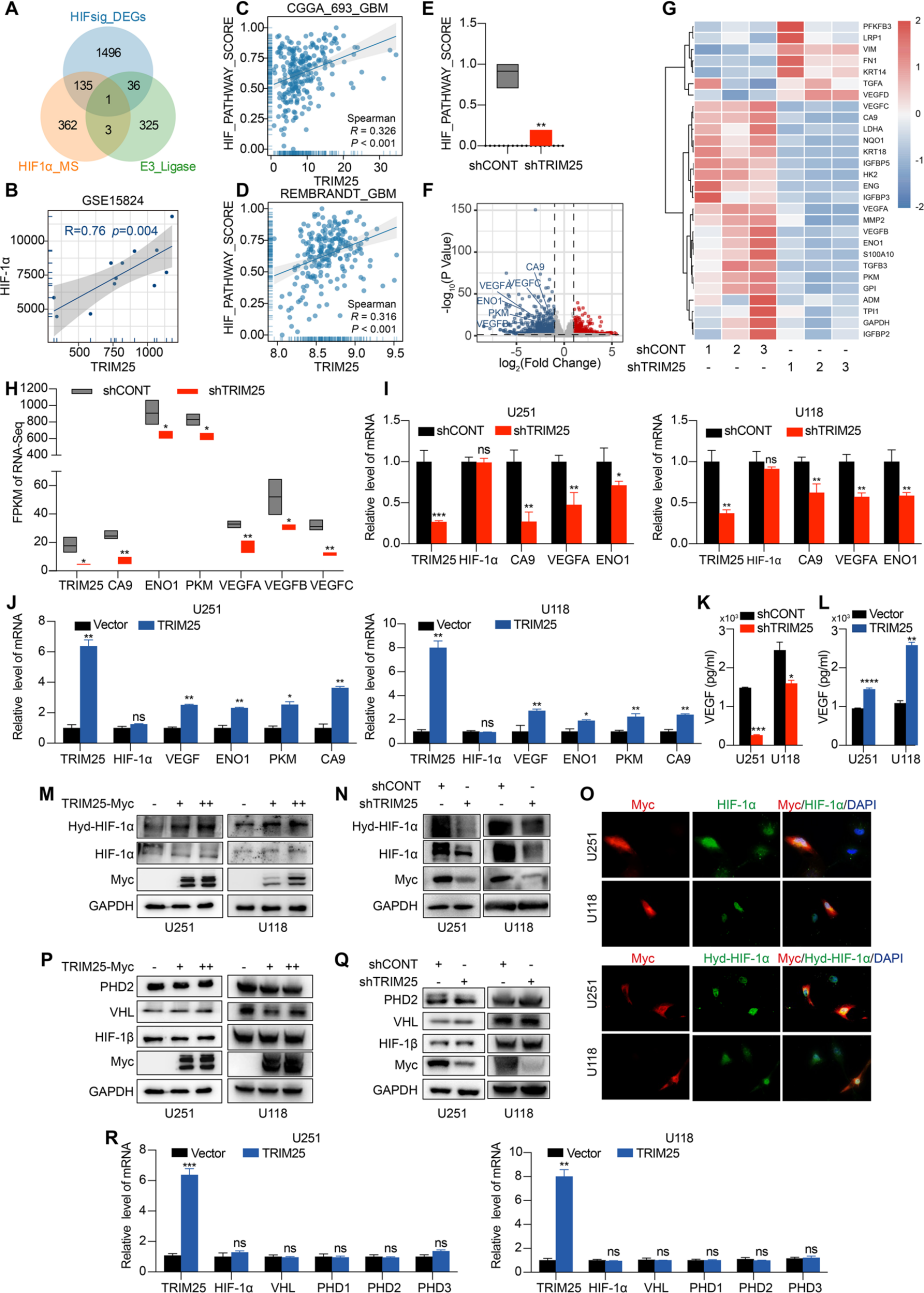
TRIM25 regulates the HIF-1 signaling pathway by promoting HIF-1α expression. **A** Venn diagram of mass spectrometry, high- and low-scoring differential genes of the HIF-1 signaling pathway under normoxia (*p* < 0.05, log fold change [FC] > 1), and the E3 ubiquitin ligase intersecting set. **B** Correlation of TRIM25 with HIF-1α expression in the GEO primary GBM cell RNA-Seq dataset (GSE15824). Correlation between TRIM25 and the HIF-1 signaling pathway. Analysis of gene expression in CGGA datasets (**C**) and Rembrandt database (**D**). **E** Enrichment score of HIF-1 signaling pathway after TRIM25 knockdown. **F** Volcano plot showing the expression of HIF-1 signaling pathway-related genes and target genes after TRIM25 knockdown. **G** Heatmap showing the expression of HIF-1 signaling pathway-related genes and target genes after TRIM25 knockdown. **H**) RNA-Seq data showing the expression of HIF-1 signaling pathway-target genes after TRIM25 knockdown in U251 cells. RT-qPCR showing the mRNA expression of HIF-1 signaling pathway-target genes after TRIM25 knockdown (**I**) and overexpression (**J**) in GBM cell lines. ELISA showing VEGF protein secretion after TRIM25 knockdown (**K**) and overexpression (**L**) in GBM cell lines. Immunoblot showing HIF-1α and hydroxylated HIF-1α protein expression after TRIM25 overexpression (**M**) and knockdown (**N**) in GBM cell lines. **O** IF staining of HIF-1α and hydroxylated HIF-1α in GBM cell lines after TRIM25 overexpression (×40-fold). Immunoblot showing VHL, PHD2, and HIF-1β protein expression after TRIM25 overexpression (**P**) and knockdown (**Q**) in GBM cell lines. **R** RT-qPCR showing *VHL*, *PHD2*, and *HIF-1β* mRNA expression after TRIM25 overexpression in GBM cell lines. *p < 0.05, **p < 0.01, ***p < 0.001.

To identify mechanisms responsible for impaired HIF-1α degradation in GBM under normoxic conditions, we performed transcriptomic analysis of 12 primary GBM cell lines. The results revealed the differential activation of the HIF-1 signaling pathway, with cells divided into high- and low-scoring groups based on the pathway signature. Primary GBM cells exhibiting the longest HIF-1α protein half-life were further subjected to proteomic profiling, which identified the E3 ubiquitin ligase TRIM25 as a potential regulator of HIF-1α stability Fig. 2A). Consistently, RNA-seq analysis showed a significant positive correlation between TRIM25 and HIF-1α expression (Fig. 2B), and bioinformatics analysis further confirmed that TRIM25 expression was positively associated with the HIF-1 signaling pathway (Fig. 2C, D). Functional experiments showed that TRIM25 knockdown resulted in lower pathway scores and downregulation of multiple HIF-1 target genes (Fig. 2E–G), whereas TRIM25 overexpression produced the opposite effects (Fig. 2H–L). At the protein level, TRIM25 overexpression increased, and its knockdown decreased both total HIF-1α and hydroxylated HIF-1α protein expression without altering the expression of other key components of the HIF-1 degradation machinery (Fig. 2M, N, P, Q). Immunofluorescence (IF) analysis further confirmed that TRIM25 overexpression in GBM cells promoted the expression and nuclear translocation of total HIF-1α and hydroxylated HIF-1α (Fig. 2O). However, RT‒qPCR analysis revealed no significant effect of TRIM25 overexpression or knockdown on *HIF-1α* mRNA levels or the transcription of its upstream regulatory genes (Fig. 2R). Collectively, these findings identify TRIM25 as a key regulator that stabilizes HIF-1α and activates HIF-1 signaling in normoxic GBM cells.

### TRIM25 promotes the stabilization of hydroxylated HIF-1α protein in GBM cells

**Fig. 3:**
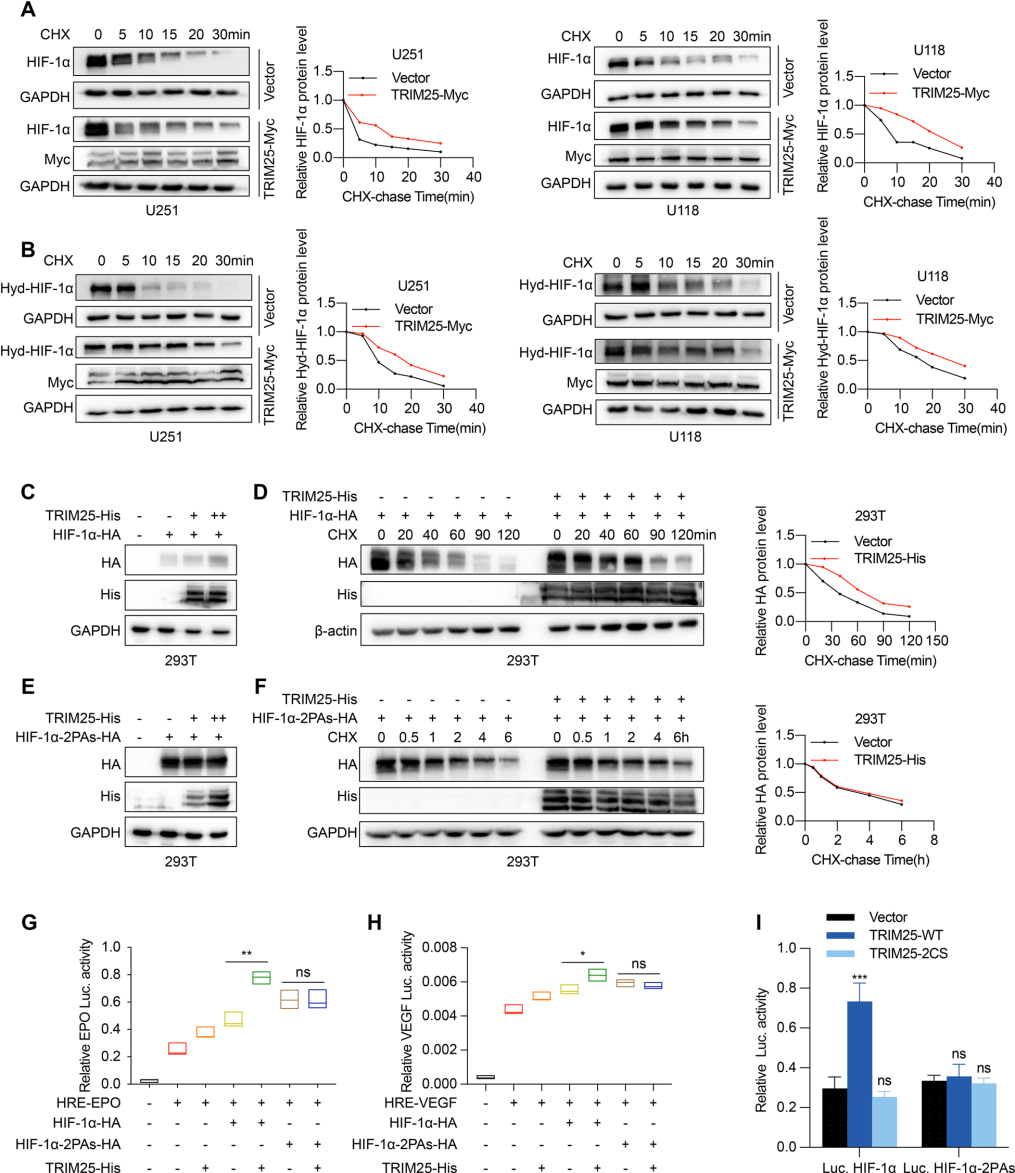
TRIM25 promotes the stabilization of hydroxylated HIF-1α protein in GBM cells. Stability of HIF-1α protein (**A**) and hydroxylated HIF-1α protein (**B**) promoted by TRIM25, as shown by western-blot assay after transfection of TRIM25-His plasmids in U251 and U118 cells treated with CHX at different incubation times. **C** HIF-1α protein expression was assessed in 293 T cells co-transfected with HIF-1α-HA and increasing amounts of the TRIM25-His plasmid. **D** HIF-1α protein expression after co-transfection of TRIM25-His and HIF-1α-HA plasmids in 293 T cells treated with CHX at different incubation times, assessed by western blot. **E** HIF-1α-2PAs protein expression after co-transfection with increasing amounts of TRIM25-His and HIF-1α-2PAs-HA plasmids in 293 T cells was assessed by western blot. **F** HIF-1α-2PAs protein expression after co-transfection of TRIM25-His and HIF-1α-2PAs-HA plasmids in 293 T cells treated with CHX at different incubation times, assessed by western blot. **G**, **H** Transcriptional regulatory activity of HIF-1α and HIF-1α-2PAs assessed after co-transfection of HRE luciferase reporter gene plasmids, His-TRIM2,5 and HA-HIF-1α or HA-HIF-1α-2PAs plasmids in 293 T cells. **I** Transcriptional regulatory activity of HIF-1α and HIF-1α-2PAs assessed by co-transfection of His-TRIM25 and His-TRIM25-2CS plasmids in 293 T cells. *p < 0.05, **p < 0.01.

To determine whether TRIM25 regulates HIF-1α protein stability, CHX chase assays were performed and the results revealed that TRIM25 overexpression significantly prolonged the half-life of both HIF-1α and hydroxylated HIF-1α proteins (Fig. 3A, B), whereas suppression of TRIM25 transcription had no significant effect on the half-life of HIF-1α mRNA (data not shown).

To determine whether this stabilizing effect was specific to the hydroxylated form of HIF-1α, a non-hydroxylatable mutant was generated by replacing proline residues 402 and 564 with alanine (A). Compared with the wild-type HIF-1α-HA. TRIM25 increased HIF-1α-HA protein expression and stability by extending its half-life but had no significant effect on HIF-1α-2PAs-HA, as confirmed by CHX chase analysis in 293 T cells (Fig. 3C–F).

Consistently, reporter assay using luciferase constructs containing the HRE sequences from the *VEGFA* and *EPO* promoter regions demonstrated that TRIM25 enhanced the transcriptional activity of HIF-1α, whereas no effect was observed for HIF-1α-2PAs (Fig. 3G–I). Together, these results suggest that TRIM25 selectively promoted HIF-1α expression by stabilizing the hydroxylated form of the protein, thereby increasing its transcriptional activity.

### TRIM25 mediates K11/29 polyubiquitination of HIF-1α at K532

**Fig. 4:**
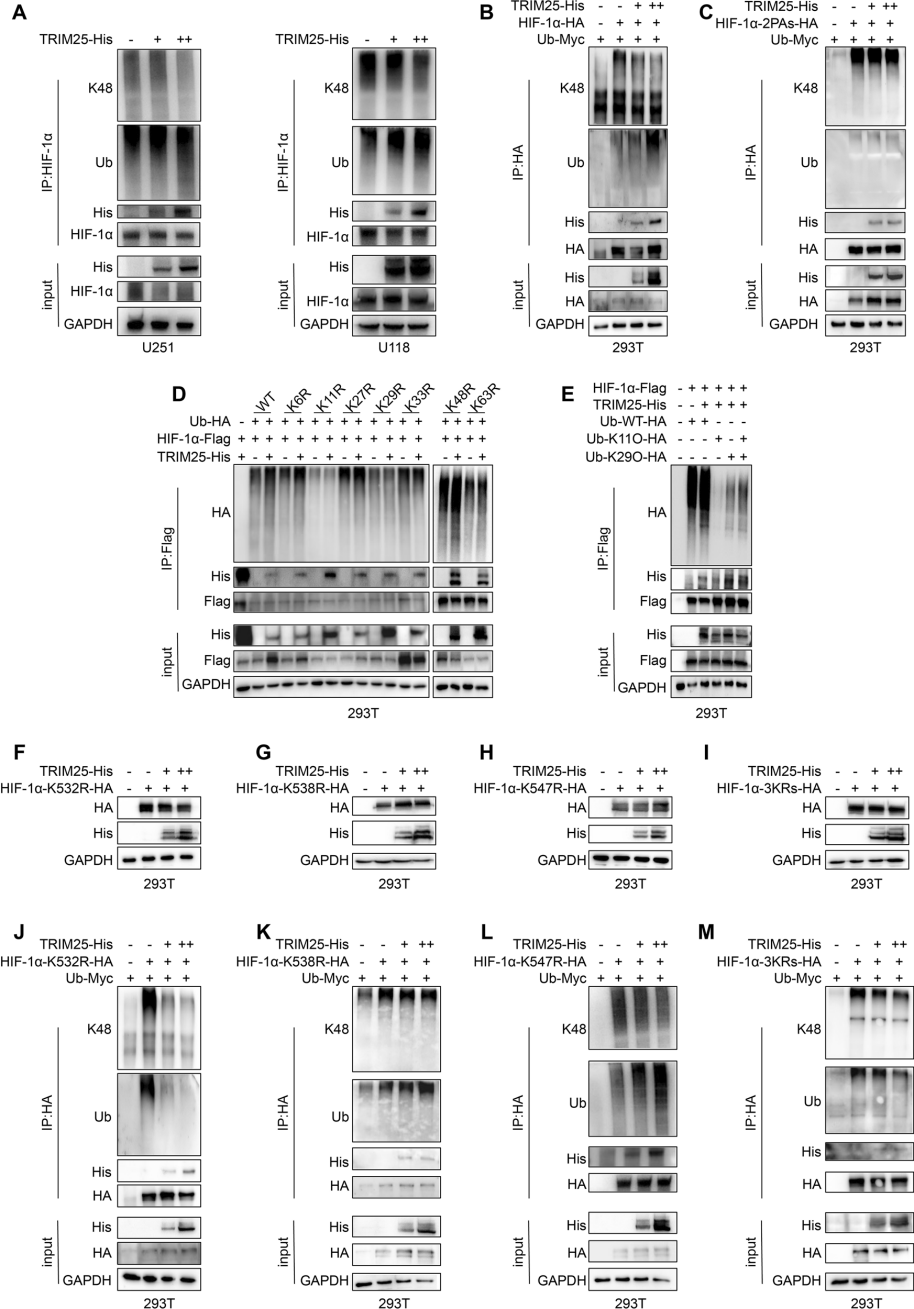
TRIM25 promotes K11/29 polyubiquitination at the K532 site of HIF-1α. **A** U251 and U118 cells were transduced with TRIM25-His, then subjected to IP with anti-HIF-1α antibody, followed by immunoblotting. **B** 293 T cells were co-transduced with HIF-1α-HA, Ub-Myc, and increasing amounts of the TRIM25-His plasmid. Cell lysates were subjected to IP using an anti-HA antibody, followed by immunoblotting. **C** 293 T cells were co-transduced with HIF-1α-2PAs-HA, Ub-Myc, and increasing amounts of the TRIM25-His plasmid. Cell lysates were subjected to IP using an anti-HA antibody, followed by immunoblotting. **D** 293 T cells were co-transduced with Ub-KxR-HA, TRIM25-His, and HIF-1α-Flag, then IP was performed with anti-Flag antibody, followed by immunoblotting. **E** 293 T cells were co-transduced with Ub-K11O-HA, Ub-K29O-HA, TRIM25-His, and HIF-1α-Flag, then IP was performed with anti-Flag antibody, followed by immunoblotting. 293 T cells were co-transduced with increasing concentrations of the TRIM25-His plasmid and HIF-1α-K532R-HA (**F**) HIF-1α-K538R-HA (**G**) HIF-1α-K547R-HA (**H**) HIF-1α-3KRs-HA (**I**), then subjected to immunoblot with antibodies against HA, His, and GAPDH. 293 T cells were co-transduced with TRIM25-His, Ub-Myc, and HIF-1α-K532R-HA (**J**) HIF-1α-K538R-HA (**K**) HIF-1α-K547R-HA (**L**) HIF-1α-3KRs-HA (**M**), then IP was performed with anti-HA antibody, followed by immunoblotting.

The effect of TRIM25 on ubiquitination was examined to elucidate the mechanism by which it promotes HIF-1α protein expression and stability. Since HIF-1α is primarily degraded through the proteasomal pathway, TRIM25 was hypothesized to influence its ubiquitination pattern. TRIM25 overexpression in GBM cells and 293 T cells increased the overall ubiquitination of HIF-1α while simultaneously reducing K48-linked polyubiquitination, the canonical signal for proteasomal degradation (Fig. 4A, B). This effect was abolished when the hydroxylation sites of HIF-1α were mutated, indicating that TRIM25-mediated modification depends on HIF-1α hydroxylation (Fig. 4C). Consistently, TRIM25 reduced the interaction between HIF-1α and the E3 ligase VHL without affecting its interaction with PHD2 (Supplementary Fig. 1A, B).

To identify the ubiquitin linkage type mediated by TRIM25, we used ubiquitin mutants in which individual lysine residues were mutated to arginine. Loss of K11 and K29 residues in 293 T cells abolished TRIM25-induced ubiquitination of HIF-1α (Fig. 4D). Complementary assay using single-lysine residue-preserving ubiquitin mutants further confirmed that TRIM25 failed to promote HIF-1α ubiquitination when ubiquitin contained only K11 or only K29, while ubiquitination was promoted when both K11 and K29 residues were present (Fig. 4E), suggesting that TRIM25 mediates K11/K29-linked polyubiquitination of HIF-1α.

Under normoxic conditions, VHL promotes K48-linked polyubiquitination degradation of hydroxylated HIF-1α at residues K532, K538, and K547 within the ODD structural domains [27, 28]. Because both TRIM25 and VHL depend on HIF-1α hydroxylation, TRIM25-mediated modification was postulated to occur at the three sites mentioned above. Mutation analyses revealed that TRIM25 retained its stabilizing effects on the HIF-1α-K538R and K547R mutants but not on the K532R mutant or the triple mutant (3KRs) (Fig. 4F–I). Moreover, TRIM25 promoted the ubiquitination of HIF-1α-K538R and HIF-1α-K547R mutants while concurrently inhibiting K48-linked polyubiquitination. Conversely, TRIM25 did not have any effect on the ubiquitination of HIF-1α-K532R or HIF-1α-3KRs (Fig. 4J–M). Overall, these findings identify K532 as the critical site through which TRIM25 mediates K11/K29-linked polyubiquitination, thereby preventing VHL-dependent K48 ubiquitination and proteasomal degradation.

### TRIM25 interacts with hydroxylated HIF-1α through the RING domain

**Fig. 5:**
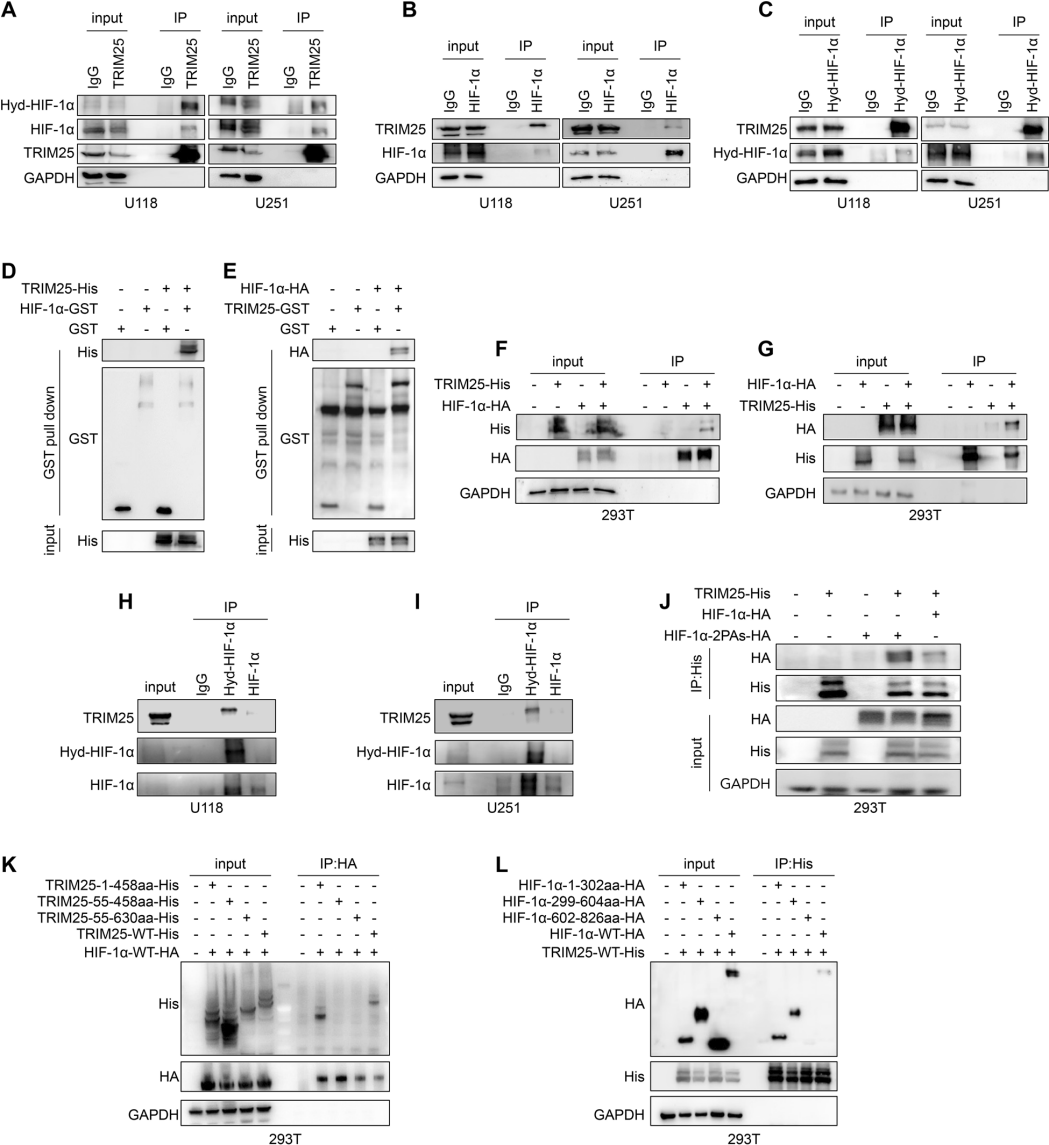
TRIM25 interacts with hydroxylated HIF-1α through the RING domain. Immunoblot of IP with anti-TRIM25 (**A**), anti- HIF-1α (**B**), anti- Hyd-HIF-1α (**C**) antibody in U251 and U118 cells. The nonspecific IgG is the control. **D**, **E** GST-pulldown assay demonstrating that TRIM25 binds to HIF-1α. 293 T cells were co-transduced with TRIM25-His and/or HIF-1α-HA, then IP was performed with anti-HA (**F**) or anti-His (**G**) antibody, followed by immunoblotting. **H**, **I** In U251 and U118 cells, IP was performed with antibodies sequentially against hydroxylated HIF-1α and HIF-1α, and the binding of TRIM25 to the hydroxylated and non-hydroxylated HIF-1α was detected by western blotting. **J** 293 T cells were co-transduced with TRIM25-His, HIF-1α-HA, and HIF-1α-2PAs-HA, then IP was performed with anti-His antibody, followed by immunoblotting. **K** Mapping of the domain of TRIM25 of its interaction with HIF-1α. 293 T cells were co-transfected with HIF-1α-HA and His-tagged TRIM25 mutants, followed by IP performed with anti-HA antibody and IB analysis with the indicated antibodies. **L** Mapping of the domain of HIF-1α of its interaction with TRIM25. 293 T cells were co-transfected with TRIM25-His and HA-tagged HIF-1α mutants, followed by IP performed with anti-His antibody and IB analysis with the indicated antibodies.

Based on proteomic evidence, TRIM25 was proposed to physically associate with HIF-1α. Co-IP experiments confirmed that TRIM25 interacted with both HIF-1α and hydroxylated HIF-1α in GBM cells and 293 T cells (Fig. 5A–C, F, G) a finding further validated by GST pull-down analysis (Fig. 5D, E). Additional co-IP experiments demonstrated that TRIM25 bound both non-hydroxylated and hydroxylated HIF-1α but showed stronger affinity for the hydroxylated form (Fig. 5H, I). Consistently, in 293 T cells expressing mutant HIF-1α lacking hydroxylation sites (HIF-1α-2PAs-HA), the binding affinity for TRIM25 was significantly reduced compared with that of wild-type HIF-1α-HA (Fig. 5J). These results indicated that TRIM25 preferentially interacted with the hydroxylated form of HIF-1α. Co-IP experiments were then performed using truncated TRIM25 mutants to identify the domains responsible for this interaction. The TRIM25 mutant containing the RING domain (residues 1–458) retained the ability to bind to HIF-1α comparable to that of full-length TRIM25, whereas deletion of this domain (residues 55–458 or 55–630) abolished this interaction (Fig. 5K, Supplementary Fig. 1C). Similarly, co-IP using truncated mutants of HIF-1α revealed that the N-terminal region (residues 1–640) mediated the binding to TRIM25, whereas the C-terminal region (residues 602–826) did not show any interaction (Fig. 5L, Supplementary Fig. 1D). This finding suggested that the RING domain of TRIM25 (residues 13–53) directly interacted with the N-terminal region of HIF-1α, which contained multiple binding interfaces distinct from those involved in VHL association near the ODD domain. This structural arrangement suggested that TRIM25 bound HIF-1α independently of hydroxylation at the ODD domain.

### TRIM25 promotes GBM progression through HIF-1α signaling

RNA-seq analysis of the 12 primary cell lines (GSE15824) revealed a positive correlation between TRIM25 expression and the HIF-1 signaling pathway (Supplementary Fig. 2A, B). Consistently, bioinformatic analysis indicated that *TRIM25* mRNA was frequently overexpressed in GBMs compared with human normal astrocytes (Supplementary Fig. 2C). IHC with a tissue microarray confirmed that TRIM25 protein expression progressively increased from grade II to IV gliomas relative to normal brain tissues (Supplementary Fig. 2D, E). Survival analysis of 60 glioma patients with detailed survival information indicated that higher TRIM25 expression was associated with poorer overall survival, independent of tumor grade compared to those with low TRIM25 expression (Supplementary Fig. 2F). Moreover, combined IHC analysis revealed a positive correlation between their expression in GBM samples (Supplementary Fig. 2G). Consistently, elevated TRIM25 expression was also observed in primary GBM cells derived from the tumor of GBM patients (Supplementary Fig. 2H, I). Collectively, these results indicate that TRIM25 expression correlates with GBM malignancy and with HIF-1α activation under normoxic conditions.

**Fig. 6:**
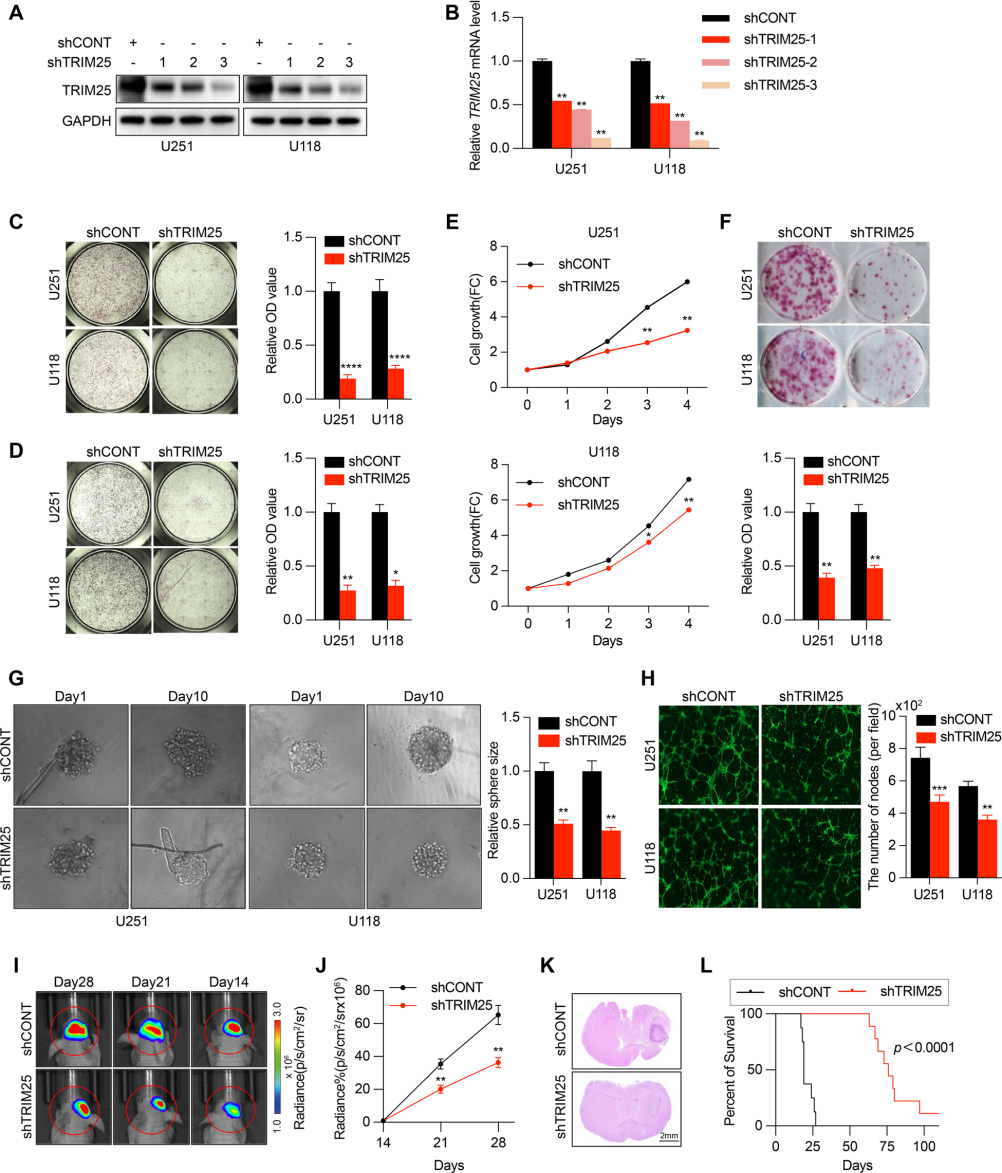
TRIM25 works as an oncoprotein in GBM cells and is associated with HIF-1α expression. **A** Immunoblot analysis of TRIM25 expression in U251 and U118 GBM cells transduced with control shRNA and shTRIM25. **B** qRT-PCR analysis of *TRIM25* mRNA expression in U251 and U118 GBM cells transduced with control shRNA and shTRIM25. **C** Transwell assays showing that TRIM25 knockdown inhibited the migration of GBM cells. **D** Transwell assays showing that TRIM25 knockdown inhibited the invasion of GBM cells. **E** CCK-8 assays showing that TRIM25 knockdown inhibited GBM cell proliferation. **F** Plate cloning assay showing that TRIM25 knockdown inhibited the clone-forming ability of GBM cells. **G** 3D tumor sphere-forming assay showing that TRIM25 knockdown inhibited the stem cell sphere-forming ability of GBM cells (×10-fold). **H** Conditioned medium derived from TRIM25 knockdown GBM cell lines inhibited vascular endothelial cell tubularity (Scale bar, 20 μm). **I** In vivo bioluminescent imaging of tumor growth performed in mice bearing GBM xenografts derived from U251 GBM cells transduced with shCONT or shTRIM25, on day 14, 21, and 28 (*n* = 6). **J** TRIM25 knockdown inhibited intracranial tumor growth in mice. **K** Representative images of H&E staining of mouse brains collected on day 28 after transplantation of U251 GBM cells transduced with shCONT or shTRIM25. Scale bar, 2 mm. **L** Kaplan–Meier survival analysis of TRIM25 knockdown in tumor-bearing mice (*n* = 8). *p < 0.05, **p < 0.01, ***p < 0.001.

To determine the functional role of TRIM25 in GBM, stable TRIM25 knockdown cell lines were established using lentiviral shRNA transfection. Among the tested constructs, shTRIM25-3 achieved the most efficient knockdown effect and was selected for subsequent experiments (Fig. 6A, B). TRIM25 knockdown significantly reduced the migration and invasion of GBM cells (Fig. 6C, D) and also decreased both cell viability and clonogenic growth (Fig. 6E, F). Second-generation 3D stem cell sphere formation experiments further revealed that TRIM25 knockdown impaired the self-renewal ability of GBM cells (Fig. 6G). Conditioned medium from TRIM25-deficient GBM cells also significantly reduced endothelial tube formation, indicating reduced angiogenic potential (Fig. 6H). In vivo experiments on subcutaneous xenografts confirmed that TRIM25 knockdown significantly inhibited tumor growth and prolonged the survival of tumor-bearing mice (Fig. 6I–L). These findings suggested that TRIM25 promoted GBM progression by enhancing proliferation, invasion, stemness, and angiogenesis. To determine whether the oncogenic function of TRIM25 depends on HIF-1α, GBM cell lines were established with TRIM25 knockdown (TRIM25-KD), HIF-1α overexpression (HIF-1α-OE), or combined TRIM25 knockdown and HIF-1α overexpression (TRIM25-KD + HIF-1α-OE) (Supplementary Fig. 3A, B). HIF-1α OE partially rescued the suppressive effect of TRIM25KD on GBM cell proliferation, invasion, and migration (Supplementary Fig. 3C–G). Consistent results were obtained in complementary in vitro studies (Supplementary Fig. 3H–I). Together, these results indicate that TRIM25 promoted GBM progression mainly through HIF-1α–dependent signaling.

### Pharmacological targeting of the TRIM25 RING domain suppresses GBM growth

**Fig. 7:**
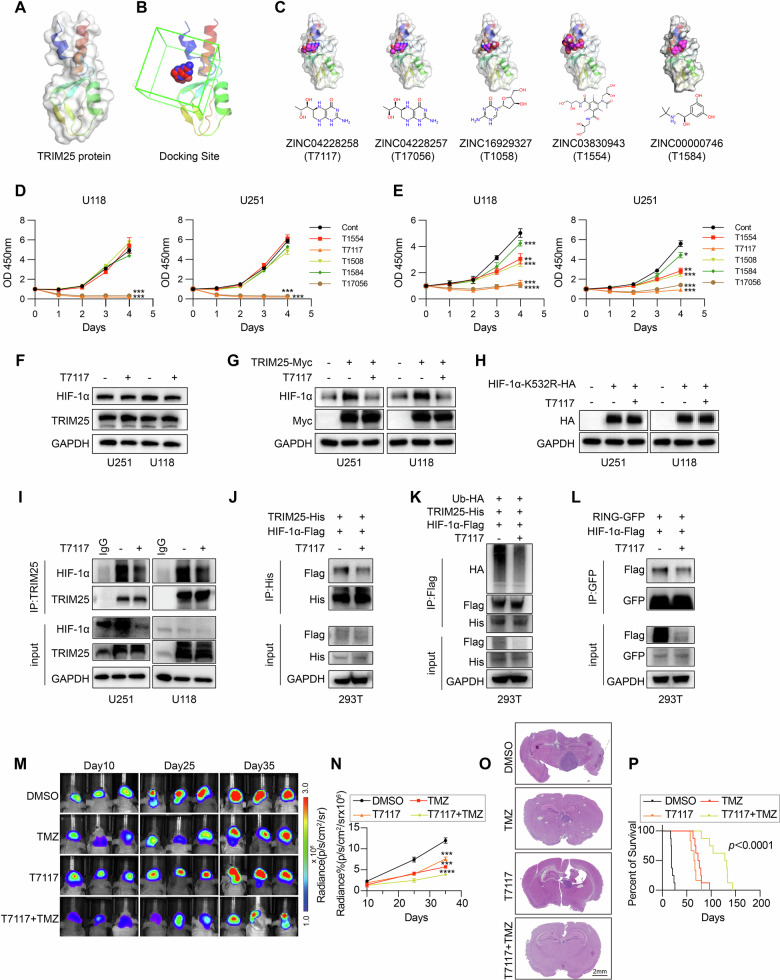
T7117 targeting the TRIM25 RING domain is an effective GBM suppressive strategy. **A** Crystal structure model of the TRIM25 protein. **B** Simulation of the RING structural domain in the binding region of TRIM25 and HIF-1α. **C** Docking model of a small molecule drug screened against the RING structural domain of the TRIM25 and HIF-1α binding region. **D** CCK-8 assay showing the proliferation of GBM cell lines after the treatment with all five compounds at a concentration of 1 mmol. **E** CCK-8 assay showing the proliferation of GBM cell lines after the treatment with all five compounds at a concentration of 10 mmol. **F** Immunoblotting showing HIF-1α protein expression after the treatment with T7117 in U251 and U118 GBM cells. **G** Immunoblotting showing HIF-1α protein expression after the transfection of TRIM25-Myc plasmids in U251 and U118 cells treated with T7117. **H** GBM cells were transfected with HIF-1α-K532R-HA, then treated with T7117, followed by immunoblotting. **I** Immunoblot of IP with anti-TRIM25, after the treatment with T7117 in U251 and U118 cells. The nonspecific IgG is the control. **J** 293 T cells were co-transfected with TRIM25-His and HIF-1α-Flag, then treated with T7117, and IP was performed with anti-His antibody, followed by immunoblotting. **K** 293 T cells were co-transfected with Ub-HA, TRIM25-His, and HIF-1α-Flag, then treated with T7117, and IP was performed with anti-Flag antibody, followed by immunoblotting. **L** 293 T cells were co-transfected with RING-GFP and HIF-1α-Flag, then treated with T7117, and IP was performed with anti-GFP antibody, followed by immunoblotting. **M**, **N** In vivo bioluminescent imaging of tumor growth performed in mice bearing GBM xenografts derived from U251 GBM cells among different treatment groups (DMSO, TMZ, T7117, or T7117 + TMZ). (*n* = 6). **O** Representative images of H&E staining of mouse brains collected on day 35 after transplantation of U251 GBM cells among different treatment groups (DMSO, TMZ, T7117, or T7117 + TMZ). Scale bar, 2 mm. **P** Kaplan–Meier survival curves of mice implanted with U251 cells among different treatment groups (DMSO, TMZ, T7117, or T7117 + TMZ). (*n* = 8). **p < 0.01, ***p < 0.001, ****p < 0.0001.

Since TRIM25 functions as an oncoprotein and its RING domain mediates the interaction with HIF-1α, the targeted inhibition of this domain was explored as a potential therapeutic approach in GBM. Virtual screening of an FDA-approved drug library identified five top-ranking candidate compounds predicted to bind the TRIM25 RING domain based on docking scores (Fig. 7A–C). Subsequent biological validation revealed that among these candidates, only T7117 and T17056 inhibited cell proliferation at a concentration of 1 mM, with all five compounds exhibiting inhibitory effects at 10 mM; however, T7117 and T17056 displayed the most significant inhibitory effects (Fig. 7D, E). Mechanistically, T7117 inhibited HIF-1α protein expression without affecting the HIF-1α-K532 mutant (Fig. 7F–H). Co-IP experiments further confirmed that T7117 disrupted the interaction between TRIM25 and HIF-1α, thereby inhibiting TRIM25-mediated ubiquitination of HIF-1α through direct interference with the RING domain (Fig. 7I–L). An intracranial xenograft model treated with an intraperitoneal injection of T7117 revealed that T7117 effectively inhibited intracranial tumor growth (Fig. 7M–O) and prolonged the survival time in mice bearing luciferase-labeled U251 tumors (Fig. 7P). Additionally, Ki-67 staining in the tumor reduced the proliferation in the combination group, indicating a suppressive effect on tumor growth (Supplementary Fig. 4A). Importantly, no significant toxicity was observed, as body weight trends remained stable, and histopathological analysis of liver, lung, and kidney from end-stage animals revealed no treatment-related toxicity following combined T7117 and TMZ administration (Supplementary Fig. 4B–E). These findings demonstrate that pharmacological inhibition of the TRIM25 RING domain with T7117 effectively disrupts TRIM25–HIF-1α signaling and inhibits GBM progression, highlighting TRIM25 as a potential therapeutic target.

## Discussion

In glioblastoma (GBM) research, HIF‑1α is widely recognized as a critical driver of malignant progression and therapeutic resistance [29–31]. Although the regulation of HIF‑1α under hypoxia and its canonical PHD‑VHL‑mediated degradation pathway have been well characterized, the mechanisms underlying HIF‑1α stabilization in normoxic GBM cells remain incompletely understood. The E3 ubiquitin ligases governing non‑canonical post‑translational modification and stability of HIF‑1α in normoxic GBM have not been clearly defined, representing a long‑standing gap in targeted therapy development. HIF‑1α in GBM can also be abnormally stabilized under normoxia via a complex regulatory network at transcriptional, translational, and post‑translational levels, enabling tumor cells to maintain malignant phenotypes even in oxygen‑replete conditions and promote progression, migration, and invasion [32–35].

**Fig. 8:**
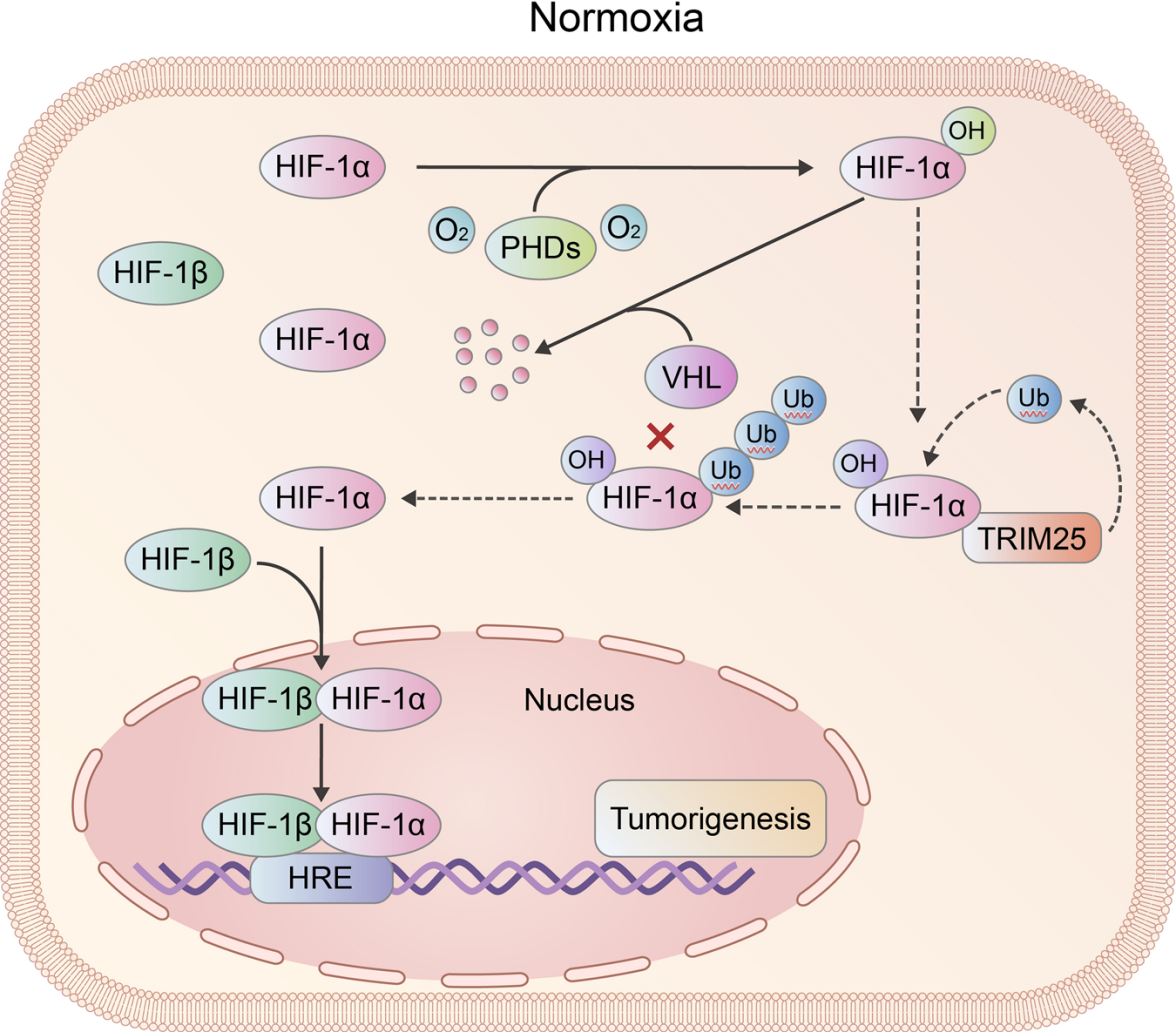
Proposed working model of the study. The E3 ubiquitin ligase TRIM25, abnormally overexpressed in GBM cells under normoxic conditions, binds to the N’-terminal peptide of HIF-1α through its RING domain, inducing K11/K29-linked nondegradative polyubiquitination at the K532 residue of hydroxylated HIF-1α. This modification stabilizes HIF-1α protein and enhances its accumulation, thereby promoting GBM progression.

In this study, we demonstrated that HIF‑1α expression and HIF pathway activity remained highly active in GBM cells even under normoxic conditions. Mechanistically, the aberrantly overexpressed E3 ligase TRIM25 bound the N‑terminal region of HIF‑1α via its RING domain and induced K11/K29‑linked polyubiquitination at the K532 site of hydroxylated HIF‑1α, thereby preventing its proteasomal degradation (Fig. 8). Unlike VHL‑dependent ubiquitination that targets HIF‑1α for degradation, TRIM25‑mediated modification stabilized HIF‑1α. Furthermore, T7117, which selectively disrupts the TRIM25‑HIF‑1α interaction, exerted potent anti‑tumor effects in GBM. These findings identify the TRIM25/HIF‑1α axis as a key driver of GBM progression and a promising therapeutic target.

Ubiquitination represents a key post-translational modification regulating numerous cellular processes [36, 37]. Ubiquitin molecules form chains through the linkage of one of their seven lysine residues (K6, K11, K27, K29, K33, K48 and K63) or the N-terminal methionine residue (M1). Ubiquitination modifications are categorized according to chain topology: (1) mono- or multi-site monoubiquitination; (2) homotypic polyubiquitination; (3) heterotypic polyubiquitination, which includes hybrids and branched chains. The K11/K29-linked polyubiquitination identified in this study belongs to the heterotypic category. Although K11- or K29-linked ubiquitin chains have traditionally been associated with proteasomal degradation [38]. Our results revealed a previously unrecognized role for K11/K29 heterotypic chains in stabilizing protein expression. This discovery suggested that branched or mixed ubiquitin linkages might exert diverse biological effects distinct from those of canonical homotypic chains. Since 28 potential branched-chain configurations exist, further characterization of heterotypic polyubiquitin architectures will be an important focus for future investigation.

TRIM25 belongs to the tripartite motif (TRIM) protein family, most possessing E3 ubiquitin ligase activity and participate in tumor progression by protein‒protein interactions and stability. TRIM25 itself ubiquitinates various substrates, including hexokinase 2 (HK2), V-domain Ig suppressor of T cell activation (VISTA), RIG-1, IRF3, and NF-κB, thereby influencing oncogenic signaling and immune responses [39–41]. Accumulating studies have established that TRIM25 acts as a crucial oncogenic E3 ubiquitin ligase in glioblastoma and drives malignant progression, chemoresistance through multiple substrate‑dependent mechanisms. TRIM25 exerts its functions mainly through K48‑ and K63‑linked ubiquitination, and NONO, VDAC2, CHKα, and CIC have been identified as its substrates [42–46]. Our study revealed a distinct mechanism by which TRIM25 promoted GBM genesis by mediating K11/K29-linked polyubiquitination at the K532 site of hydroxylated HIF-1α, thereby directly stabilizing HIF-1α. Functional rescue experiments further demonstrated that HIF-1α overexpression largely reversed the inhibitory effect of TRIM25 knockdown on GBM cell proliferation and migration, suggesting that the TRIM25-HIF-1α signaling pathway represented a major oncogenic route mediated by TRIM25 in GBM.

Given the crucial role of HIF-1α, targeting this molecule holds broad application prospects. Currently available HIF‑1α inhibitors are associated with severe adverse drug reactions that prevent their advancement into subsequent clinical trials, as well as a lack of specificity, leading to unintended outcomes. T7117, also known as 6R-BH4 dihydrochloride, is a synthetic form of tetrahydrobiopterin (BH4) that has previously been approved for studies in phenylketonuria [47, 48]. In the present study, T7117 was repurposed to target the interaction between TRIM25 and HIF-1α in GBM. This compound effectively disrupted this interaction and, when combined with TMZ, markedly improved the survival of tumor-bearing mice. Drug repurposing offers notable advantages, particularly the availability of established safety data [49, 50]. Long-term treatment with T7117 in phenylketonuria patients is well tolerated, supporting, to some extent, its safety profile. However, the current study was primarily based on established GBM cell lines, which cannot fully recapitulate the complex pathological milieu, cellular heterogeneity, tumor microenvironment, and genetic background of clinical GBM tissues in patients. Further functional and toxicological studies will be required to evaluate the long-term safety and efficacy of this combination in preclinical and clinical settings.

Although this study identified a new mechanism underlying the post-translational modification of HIF-1α, several limitations should be acknowledged: This study did not distinguish the distinct roles of GBM cells in malignant progression under normoxic and hypoxic conditions or determine whether such differences depend on HIF‑1α signaling. It also remains unclear whether TRIM25 regulates other hypoxia‑associated pathways or whether TRIM25‑mediated HIF‑1α ubiquitination is controlled by additional factors. Furthermore, despite the necessity of immunodeficient mouse models in GBM research, interspecies differences in the blood–brain barrier, drug metabolism, and angiogenesis limit their ability to accurately mimic human brain drug delivery and therapeutic responses. Addressing these limitations in future studies will be essential to further elucidate the mechanisms by which TRIM25 regulates GBM pathophysiology and to assess its potential as a therapeutic target.

In summary, our study identifies TRIM25 as a critical regulator of HIF-1α stability under normoxic conditions and uncovers a non-canonical ubiquitin mechanism that sustains HIF-1 signaling in GBM. By revealing the TRIM25/HIF-1α axis as both a mechanistic driver of GBM pseudohypoxia and a druggable vulnerability, our findings provide new insights into GBM biology and open potential avenues for targeted therapeutic intervention.

## Supplementary information

Supplementary Figs. 1-4 and legends

Supplementary Table 1

Supplementary Table 2

Supplementary Table 3

Original Data

## Data Availability

The RNA-seq dataset was deposited in the Sequence Read Archive (SRA) repository at NCBI under the accession number PRJNA1347646. The datasets used and analyzed during the current study are available from the corresponding author upon reasonable request.

## Code availability

Public R packages used in this study include DESeq2 and edgeR. In-house Perl scripts for raw FASTQ sequencing data trimming are available from the corresponding author upon reasonable request, with no restrictions on access.
