# Supplementary material for: TRIM25 promotes glioblastoma progression by stabilizing HIF-1α expression in normoxia through K11/K29 polyubiquitination
Source: Cell Death Dis. 2026 Apr 22;17(1):530. doi: 10.1038/s41419-026-08757-3 (PMC13230578; doi:10.1038/s41419-026-08757-3)
Supplement: Supplementary file 1 — Supplementary Figs. 1-4 and legends [file 41419_2026_8757_MOESM1_ESM.docx]

**Supplementary Figure 1**


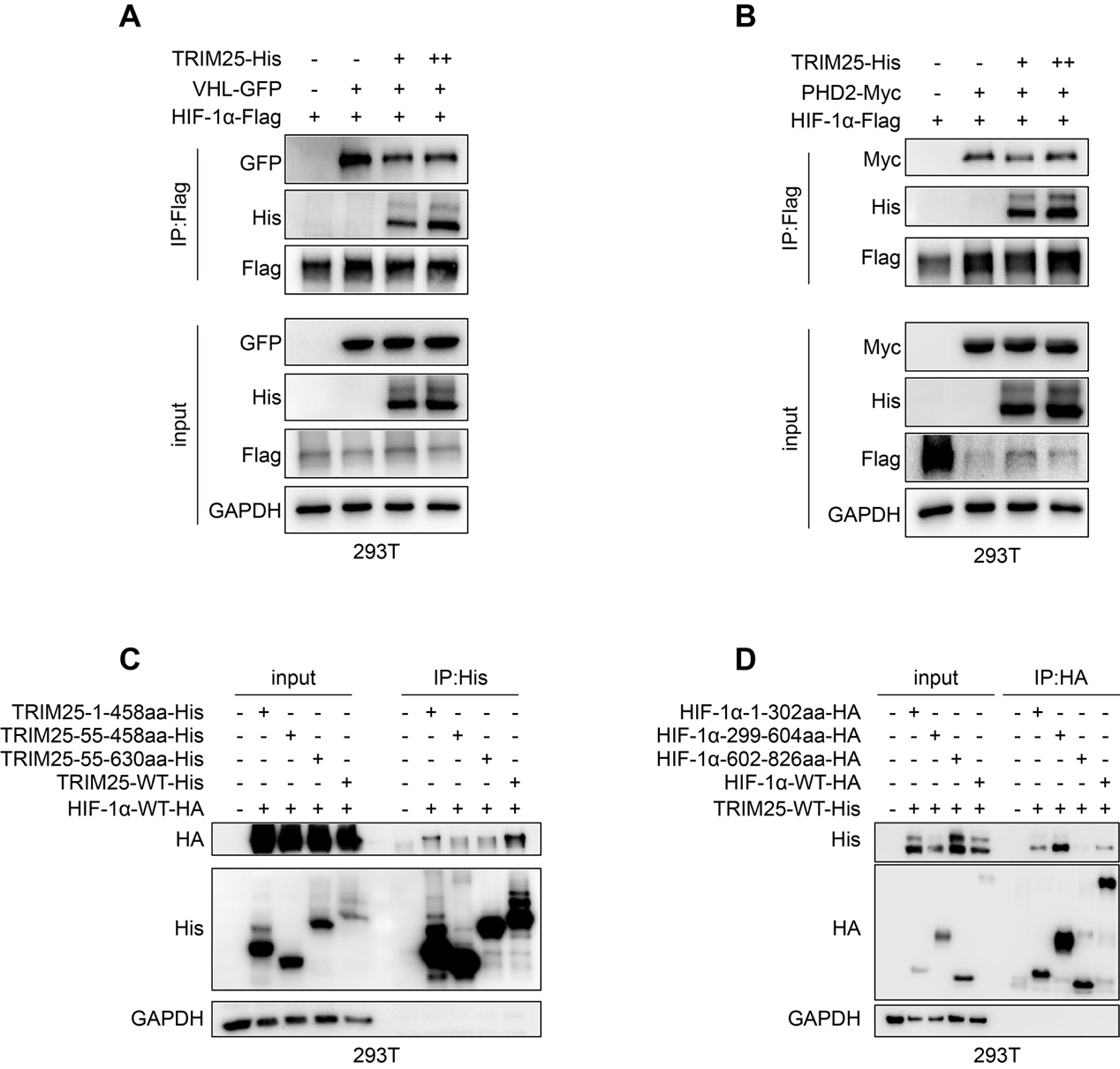


**Supplementary Figure.1| TRIM25 binds to the N-terminus of hydroxylated HIF-1α via the RING domain and reduces the binding of HIF-1α to VHL.**

**A)** 293T cells were co-transduced with HIF-1α-Flag, TRIM25-His (setting a concentration gradient) and VHL-GFP, then IP with anti-Flag antibody, followed by immunoblotting. **B)** 293T cells were co-transduced with HIF-1α-Flag, TRIM25-His (setting a concentration gradient) and PHD2-Myc, then IP with anti-Flag antibody, followed by immunoblotting. **C)** Mapping of the domain of TRIM25 for its interaction with HIF-1α. 293T cells were co-transfected with HIF-1α-HA and His-tagged TRIM25 mutants, followed by IP with anti-His antibody and IB analysis with indicated antibodies. **D)** Mapping of the domain of HIF-1α for its interaction with TRIM25. 293T cells were co-transfected with TRIM25-His and HA-tagged HIF-1α mutants, followed by IP with anti-HA antibody and IB analysis with indicated antibodies.

**Supplementary Figure 2**


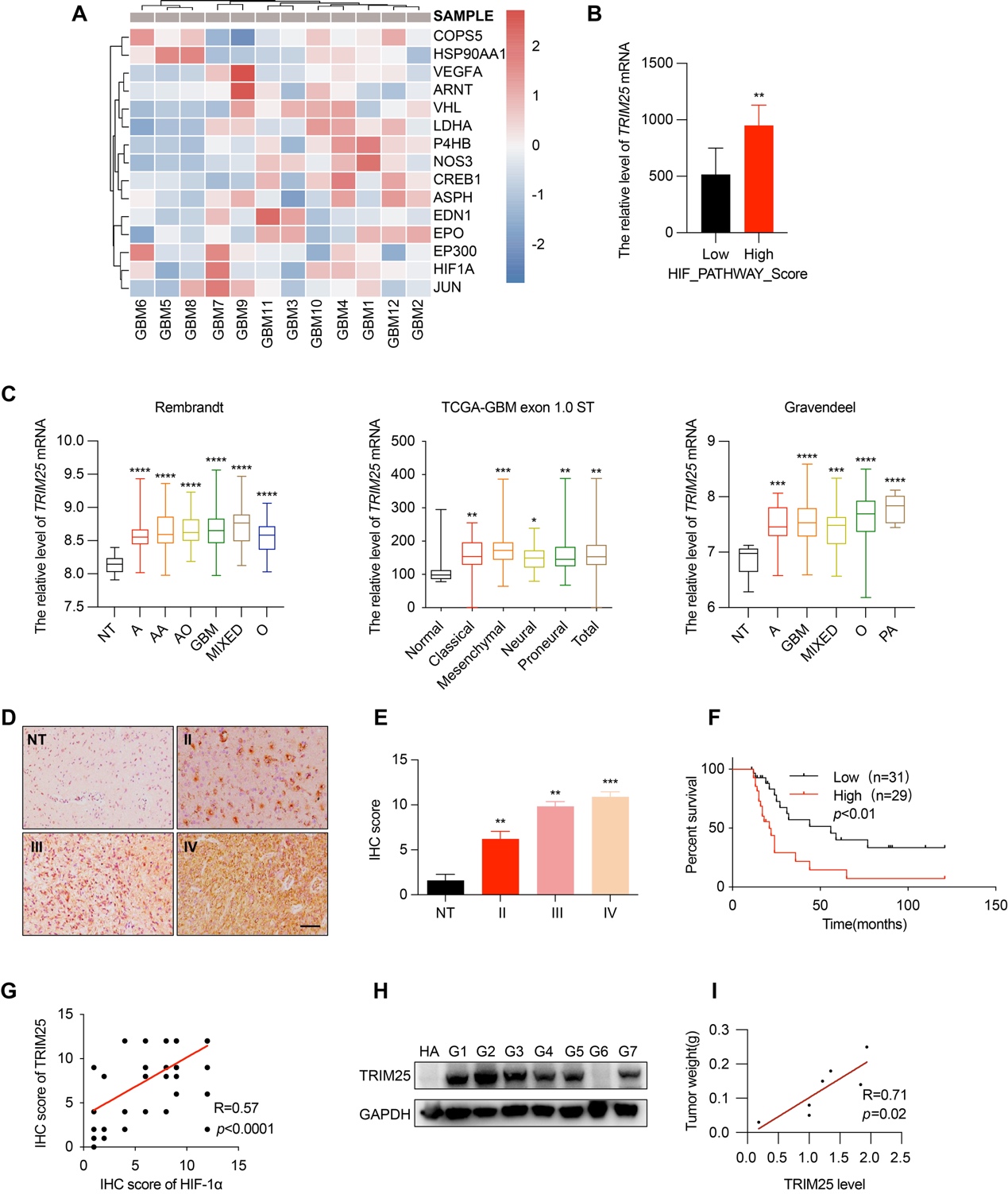


**Supplementary Figure.2| High HIF-1α expression in GBM is associated with TRIM25 expression.**

**A)** Heatmap of the expression of key molecules and target genes of the HIF-1 signaling pathway in GBM primary cell lines under normoxia. **B)** TRIM25 expression in high and low scoring groups of the HIF-1 signaling pathway in normoxia. **C)** Rembrandt, TCGA, and Gravendeel databases analyzed TRIM25 expression in different types of gliomas. **D)** Schematic representation of TRIM25 IHC resulted in different grades of glioma tissue microarrays (Scale bars, 20 μm). **E)** Expression of TRIM25 in different grades of gliomas. (NT: 5 cases, grade II: 14 cases, grade III: 20 cases, grade IV: 21 cases). **F)** Kaplan–Meier survival analysis of TRIM25. (Cut-off: mean value of tumor expression). **G)** Scatterplot of IHC score correlation between TRIM25 and HIF-1α. **H)** Expression of TRIM25 protein levels in 7 GBM primary cell lines under normoxia. **I)** Statistical analysis of the correlation between the expression level of TRIM25 and tumorigenic ability in GBM primary cell lines. **p*<0.05, ***p*<0.01, ****p*<0.001.

**Supplementary Figure 3**

**
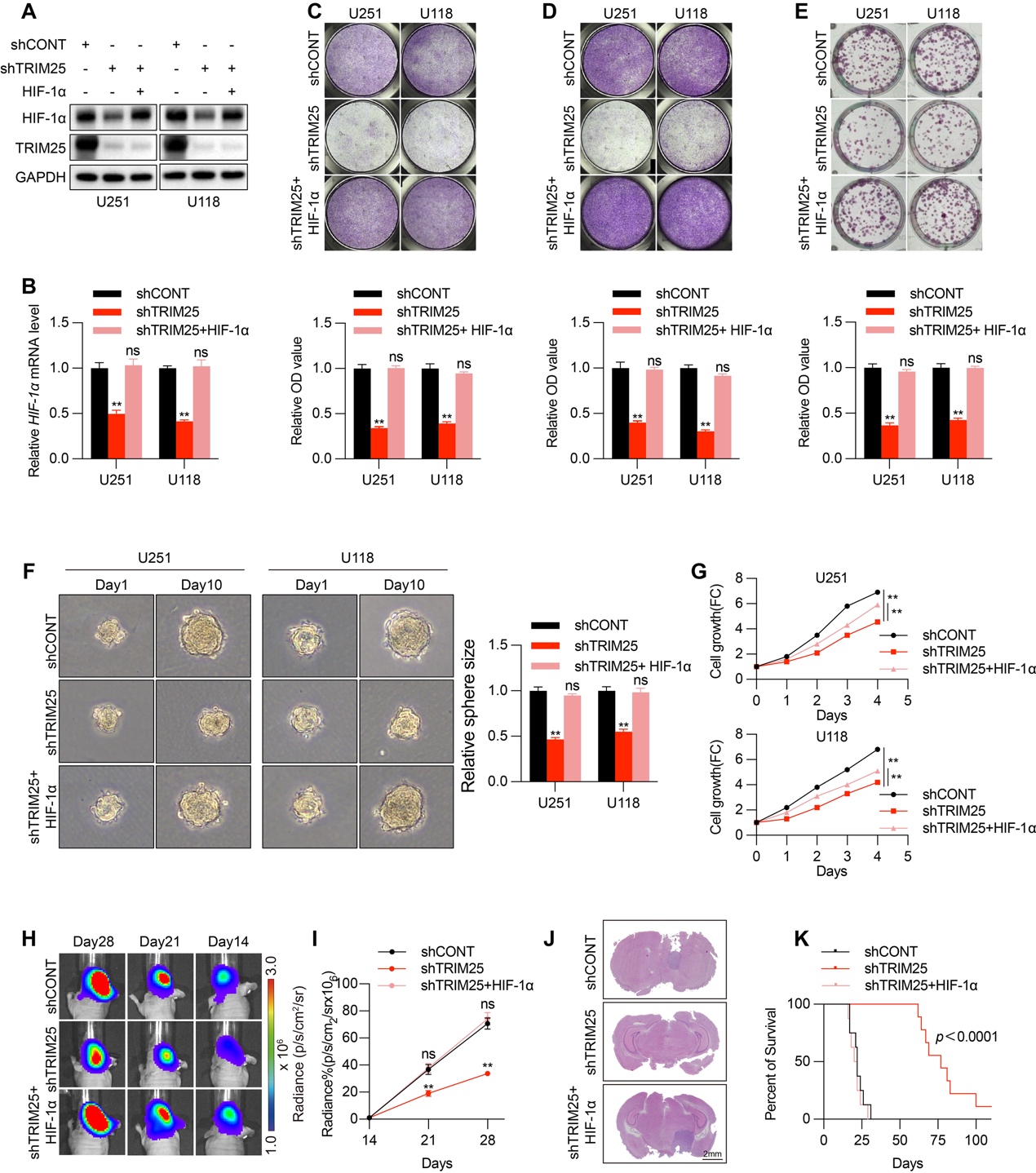
**

**Supplementary Figure.3|. The function of TRIM25 as an oncoprotein in GBM cells is associated with HIF-1α expression.**

**A)** Immunoblot analysis of TRIM25 levels in U251 and U118 GBM cells transduced with control shRNA/shTRIM25 and HIF-1α. **B)** qRT-PCR analysis of TRIM25 mRNA expression in U251 and U118 GBM cells transduced with control shRNA/shTRIM25 and HIF-1α. **C)** Transwell assays showed that the ability of TRIM25 knockdown to inhibit the migration of GBM cells was related to the expression of HIF-1α. **D)** Transwell assays showed that the ability of TRIM25 knockdown to inhibit the invasion of GBM cells was related to the expression of HIF-1α. **E)** Plate cloning assays demonstrated that the ability of TRIM25 knockdown to inhibit the clone-forming of GBM cells was related to the expression of HIF-1α. **F)** 3D tumor sphere-forming assays demonstrated that the ability of TRIM25 knockdown to inhibit the stem cell sphere-forming of GBM cells was related to the expression of HIF-1α (×10-fold). **G)** CCK-8 assays showed that the ability of TRIM25 knockdown to inhibit proliferation of GBM cells was related to the expression of HIF-1α. **H)** *In vivo* bioluminescent imaging of tumor growth was performed in mice bearing GBM xenografts derived from U251 GBM cells transduced with shCONT or shTRIM25 and HIF-1α, on days 14, 21, and 28 (*n* = 6). **I)** Statistical graph showed that TRIM25 knockdown to inhibit intracranial tumor growth in mice was related to the expression of HIF-1α. **J)** Representative images of H&E staining of mouse brains collected on day 28 after transplantation of U251 GBM cells transduced with shCONT or shTRIM25 and HIF-1α. Scale bar, 2 mm. **K)** Kaplan–Meier survival curves of mice implanted with U251 cells across different treatment groups, including shCONT, shTRIM25 and shTRIM25+HIF-1α (*n* = 8). **p*<0.05, ***p*<0.01.

**Supplementary Figure 4**


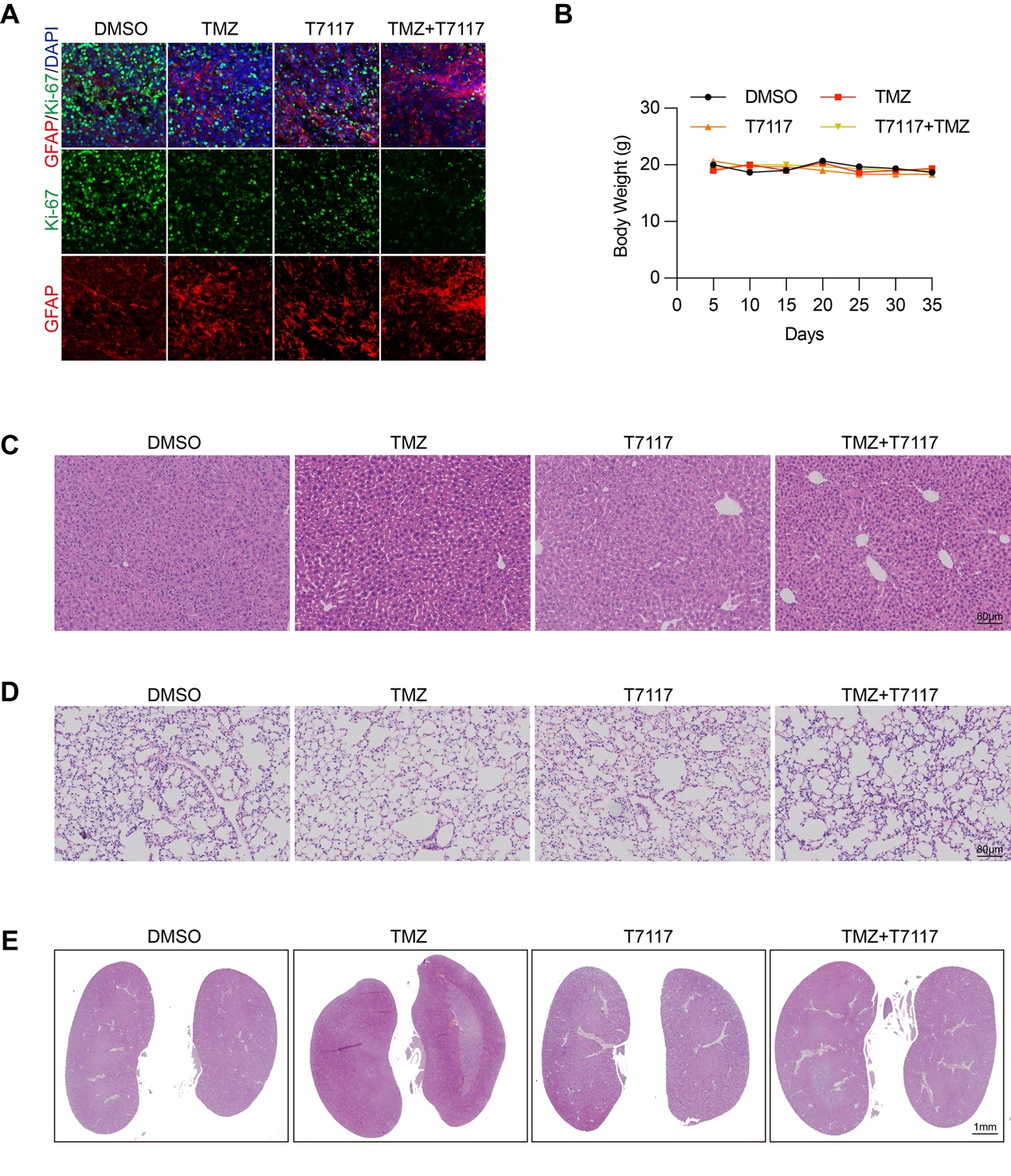


**Supplementary Figure.4|. The effect of combined administration of T7117 and TMZ on tumor-bearing mice.**

**A)** Cellular IF showing Ki-67 expression in tumors formed by GBM primary cell lines (×20-fold). **B)** The body weight monitoring for mice treated with T7117 alone and in combination with TMZ. (*n=6*). **C-E)** Representative images of H&E staining of mouse livers (C), lungs (D) and kidneys (E) collected on days 35 after transplantation of U251 GBM cells across different treatment groups, including DMSO, TMZ, T7117 or T7117+TMZ.
