# Supplementary material for: TRIM25 promotes glioblastoma progression by stabilizing HIF-1α expression in normoxia through K11/K29 polyubiquitination
Source: Cell Death Dis. 2026 Apr 22;17(1):530. doi: 10.1038/s41419-026-08757-3 (PMC13230578; doi:10.1038/s41419-026-08757-3)
Supplement: Supplementary file 2 — Supplementary Table 1 [file 41419_2026_8757_MOESM2_ESM.docx]

**Supplementary Table 1. The antibodies and dilution used to detect the indicated proteins.**

| **Antigen** | **Primary Antibody** | **Dilution** |
| --- | --- | --- |
| HIF-1α | Cell Signaling Technology; 36169; rabbit monoclonal | 1:500 for IF/IHC; 1:1000 for WB; 1:100 for IP |
| TRIM25 | Abcam; ab167154; rabbit monoclonal | 1:500 for IF/IHC; 1:1000 for WB; 1:100 for IP |
| Hydroxy-HIF-1α | Cell Signaling Technology; 3434; rabbit monoclonal | 1:500 for IF/IHC; 1:1000 for WB; 1:100 for IP |
| GFAP | Abcam; ab68428; rabbit monoclonal | 1:500 for IF/IHC |
| Ki-67 | Abcam; ab279653; mouse monoclonal | 1:500 for IF |
| PHD2 | Cell Signaling Technology; 4835; rabbit monoclonal | 1:1000 for WB |
| VHL | Cell Signaling Technology; 68547; rabbit monoclonal | 1:1000 for WB |
| HIF-1β | Cell Signaling Technology; 5537; rabbit monoclonal | 1:1000 for WB |
| Ubiquitin | Cell Signaling Technology; 3639; mouse monoclonal | 1:1000 for WB |
| K48 | Cell Signaling Technology; 8081; rabbit monoclonal | 1:1000 for WB |
| GAPDH | Proteintech; 60004-1-Ig; mouse monoclonal | 1:10000 for WB |
| β-actin | Proteintech; 66009-1-Ig; mouse monoclonal | 1:10000 for WB |
| Normal Rabbit IgG | Abcam; ab172730; rabbit monoclonal | 1:100 for IP |
| HA-Tag | Abcam; ab9110; rabbit polyclonal | 1:5000 for WB; 1:200 for IP |
| Flag-Tag | Abmart; M20008; mouse monoclonal | 1:5000 for WB; 1:200 for IP |
| His-Tag | Proteintech; 66005-1-Ig; mouse monoclonal | 1:5000 for WB; 1:200 for IP |
| GFP-Tag | Abcam; ab290; rabbit polyclonal | 1:2000 for WB |
| Myc-Tag | Cell Signaling Technology; 2276; mouse monoclonal | 1:1000 for WB |
| GST | Cell Signaling Technology; 2624; mouse monoclonal | 1:1000 for WB; 1:100 for IP |
