# Supplementary material for: TRIM25 promotes glioblastoma progression by stabilizing HIF-1α expression in normoxia through K11/K29 polyubiquitination
Source: Cell Death Dis. 2026 Apr 22;17(1):530. doi: 10.1038/s41419-026-08757-3 (PMC13230578; doi:10.1038/s41419-026-08757-3)
Supplement: Supplementary file 4 — Supplementary Table 3 [file 41419_2026_8757_MOESM4_ESM.docx]

**Supplementary Table 3. The sequences of the primers used in the study.**

| Primer | Forward (5’-3’) | Reverse (5’-3’) |
| --- | --- | --- |
| *TRIM25* | AATCGGCTGCGGGAATTTTTC | TCTCACATCATCCAGTGCTCT |
| *HIF-1α* | GAACGTCGAAAAGAAAAGTCTCG | CCTTATCAAGATGCGAACTCACA |
| *VEGFA* | AGGGCAGAATCATCACGAAGT | AGGGTCTCGATTGGATGGCA |
| *ENO1* | AAAGCTGGTGCCGTTGAGAA | GGTTGTGGTAAACCTCTGCTC |
| *PKM* | ATGTCGAAGCCCCATAGTGAA | TGGGTGGTGAATCAATGTCCA |
| *CA9* | GGATCTACCTACTGTTGAGGCT | CATAGCGCCAATGACTCTGGT |
| *VHL* | GCAGGCGTCGAAGAGTACG | CGGACTGCGATTGCAGAAGA |
| *PHD1* | TGGCCCTGGACTATATCGTG | GGCACCAATGCTTCGACAG |
| *PHD2* | GAAGGCGAACCTGTACCCC | TTCATGCACGGCACGATGTA |
| *PHD3* | CTGGGCAAATACTACGTCAAGG | GACCATCACCGTTGGGGTT |
