# Supplementary material for: TRIM25 promotes glioblastoma progression by stabilizing HIF-1α expression in normoxia through K11/K29 polyubiquitination
Source: Cell Death Dis. 2026 Apr 22;17(1):530. doi: 10.1038/s41419-026-08757-3 (PMC13230578; doi:10.1038/s41419-026-08757-3)

Supplementary File. The uncropped western blot images shown in the study.

Figure 1F

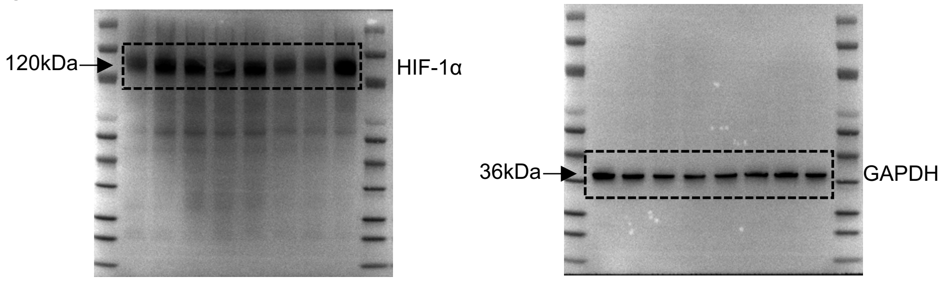

Figure 1K

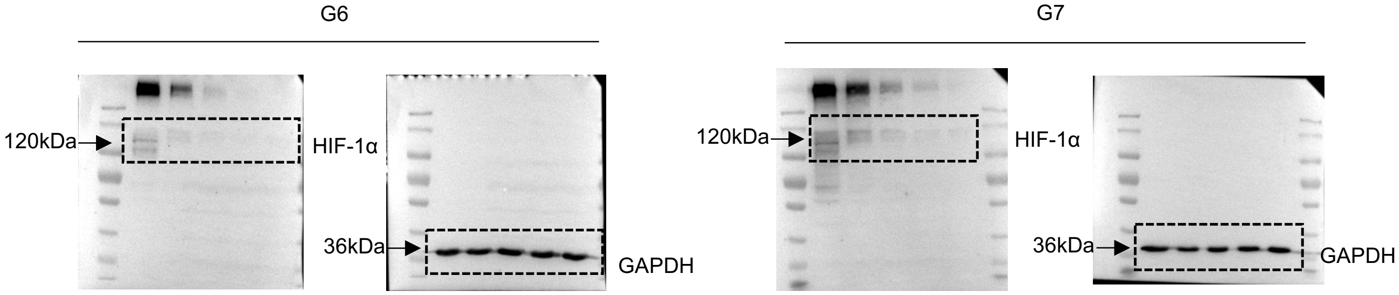

Figure 2M 2P U251

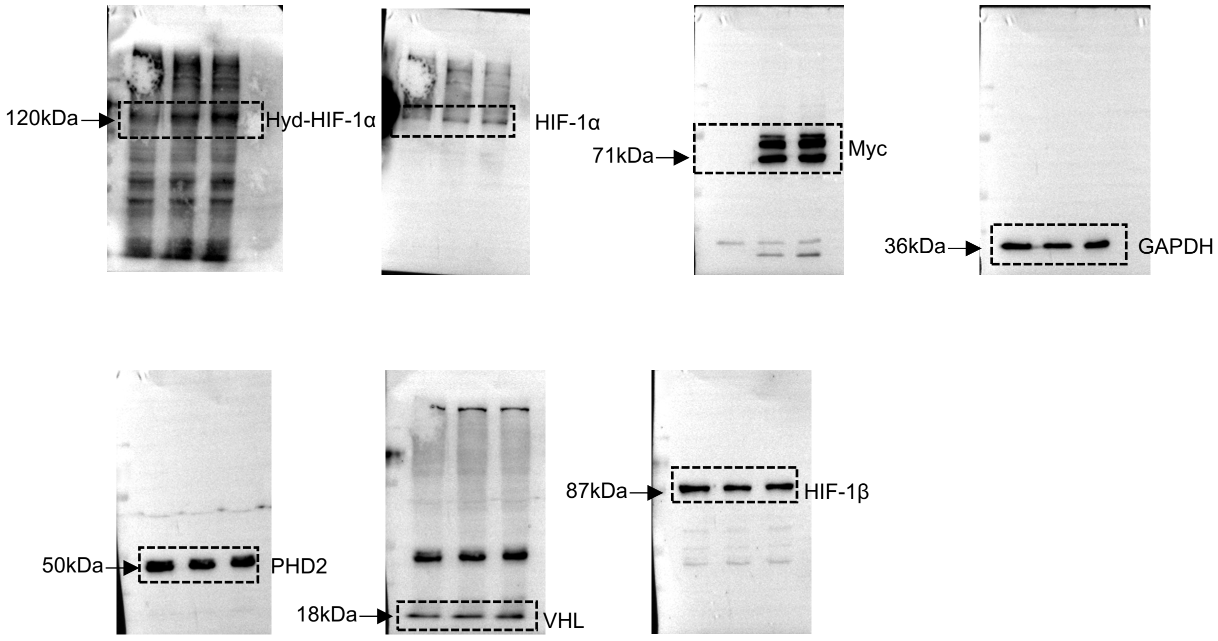

Figure 2M U118

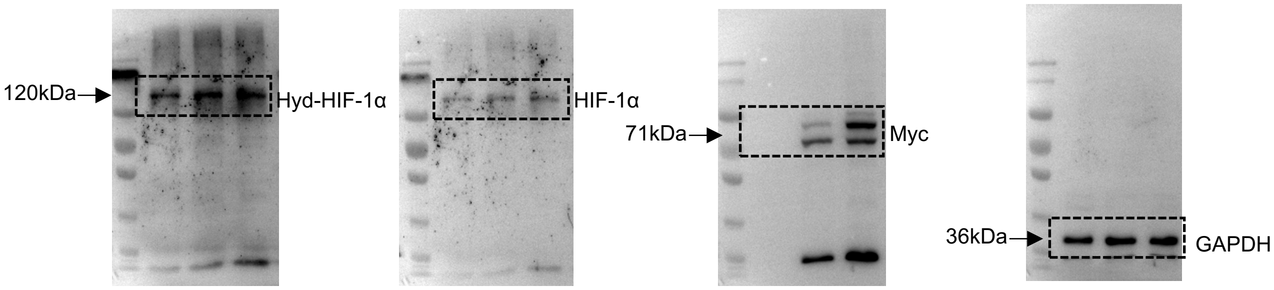

Figure 2N U251

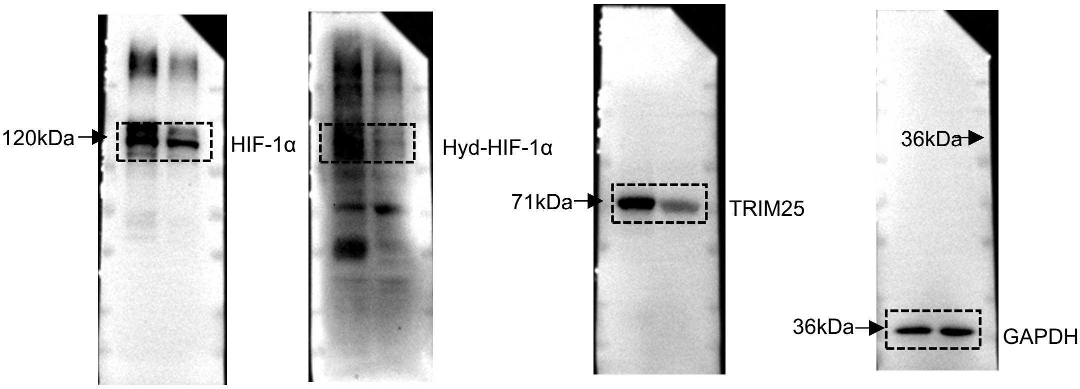

Figure 2N U118

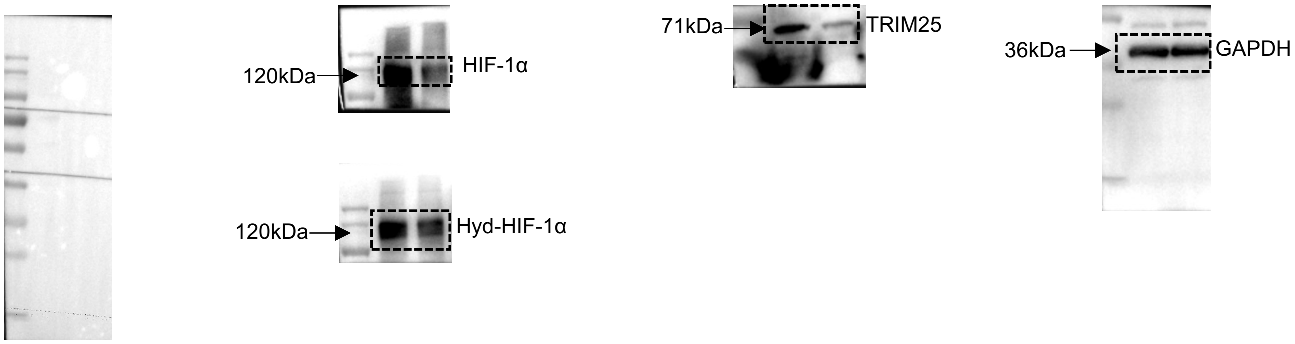

Figure 2P U118

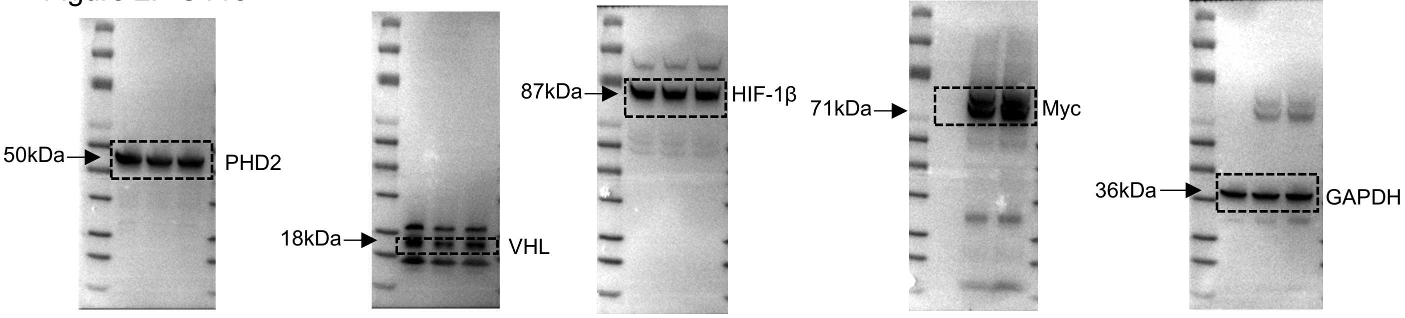

Figure 2Q U251

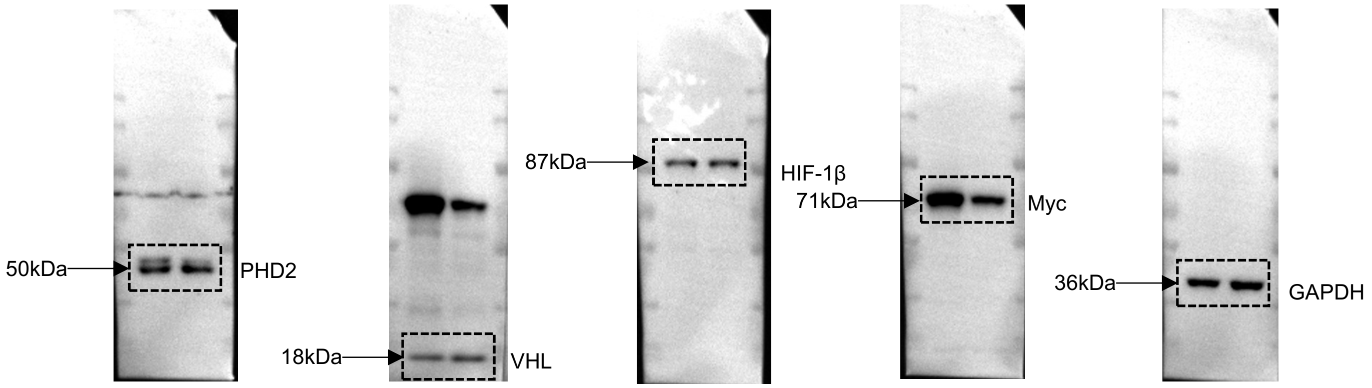

Figure 2Q U118

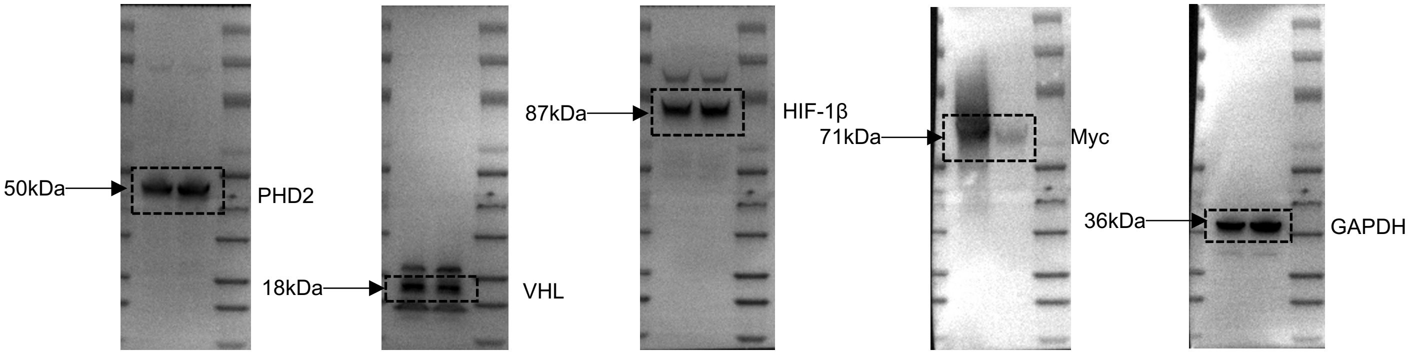

Figure 3A U251

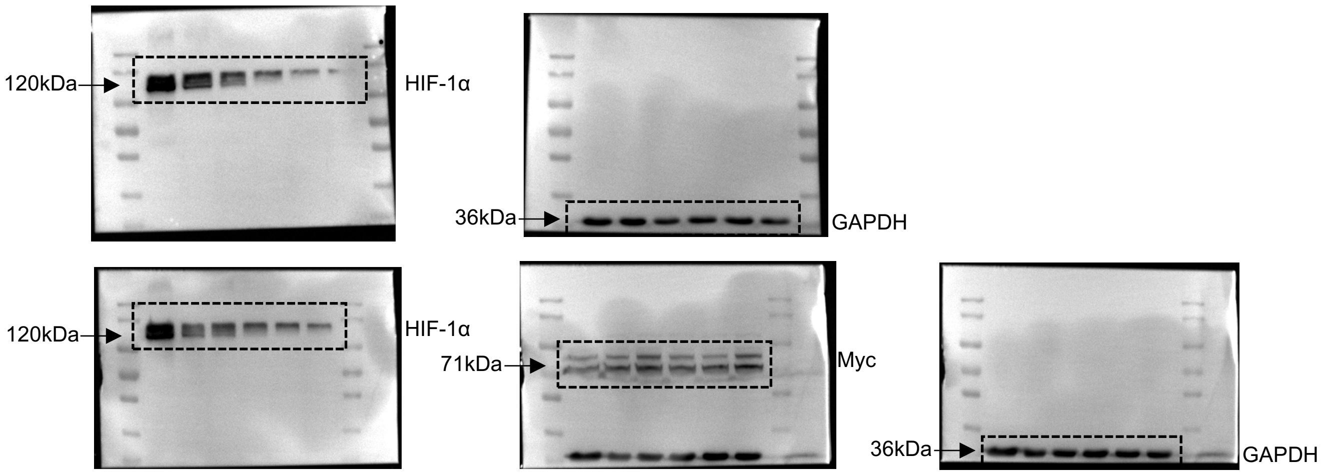

Figure 3A U118

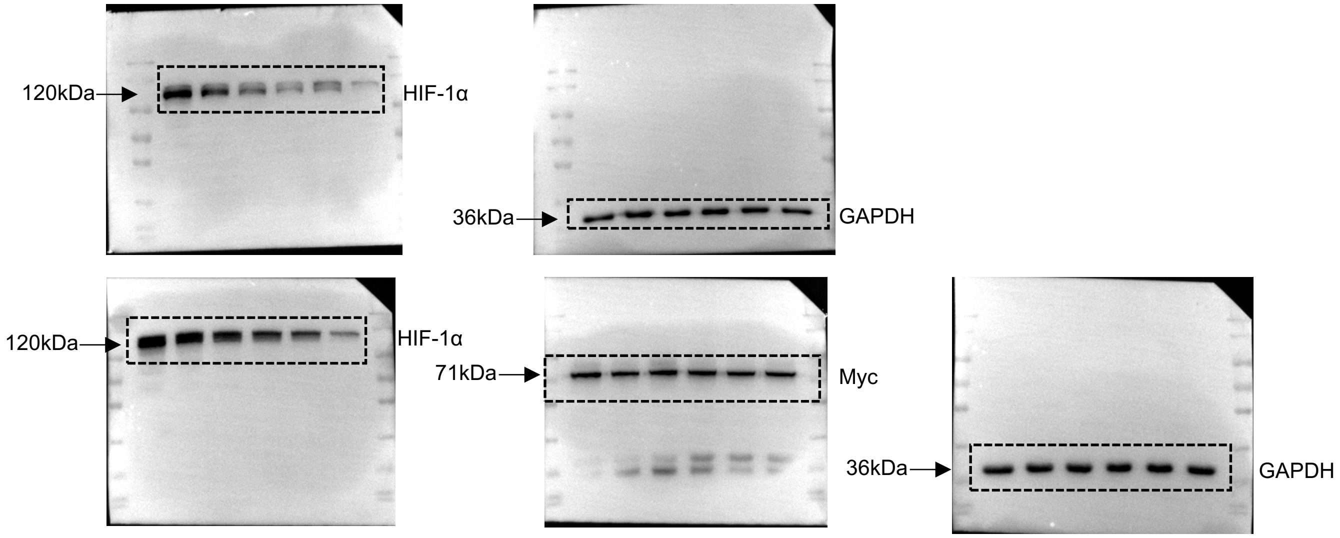

Figure 3B U251

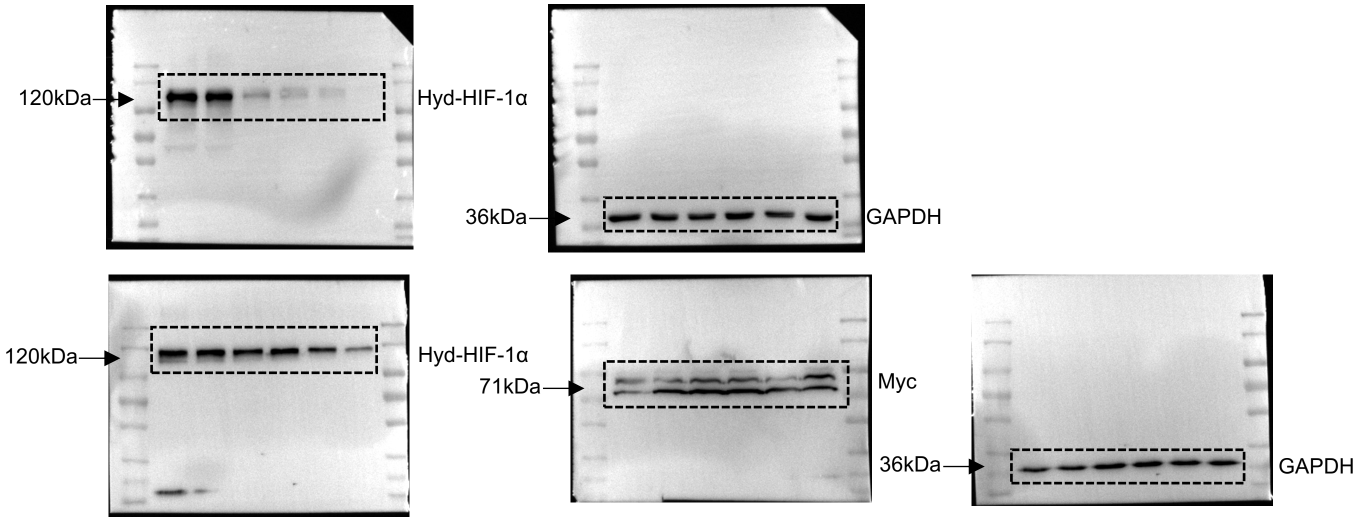

Figure 3B U118

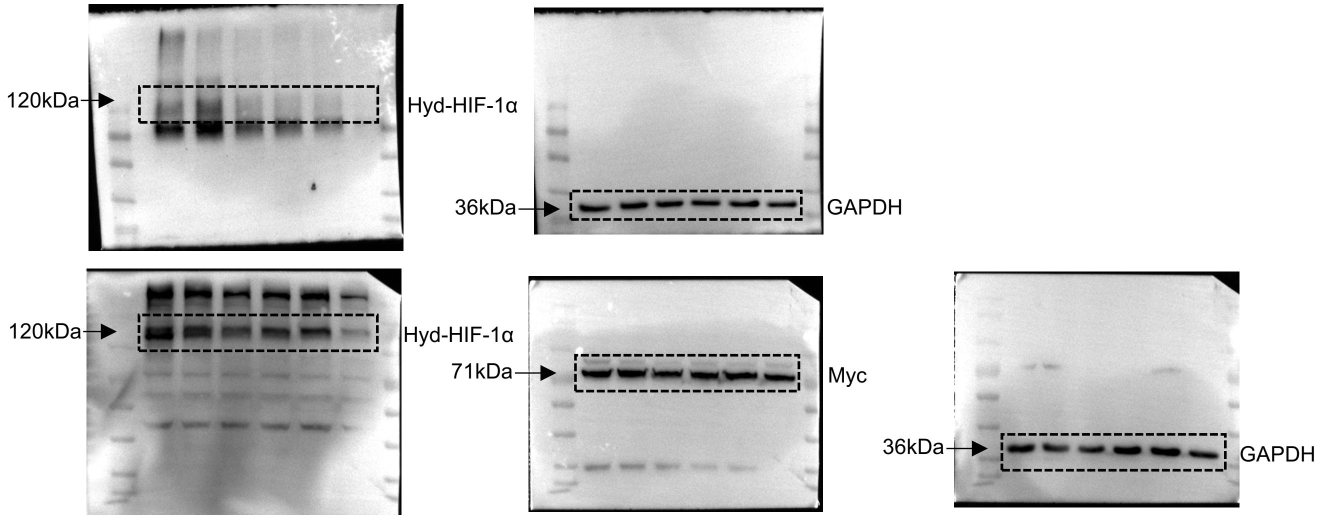

Figure 3C(left)、3E(right)

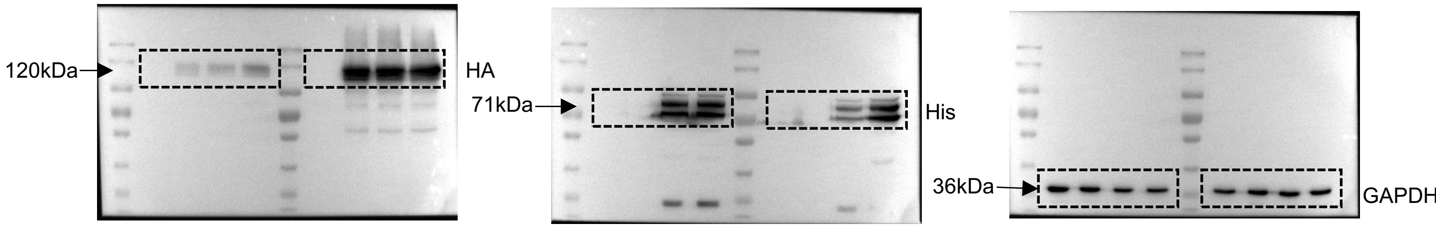

Figure 3D

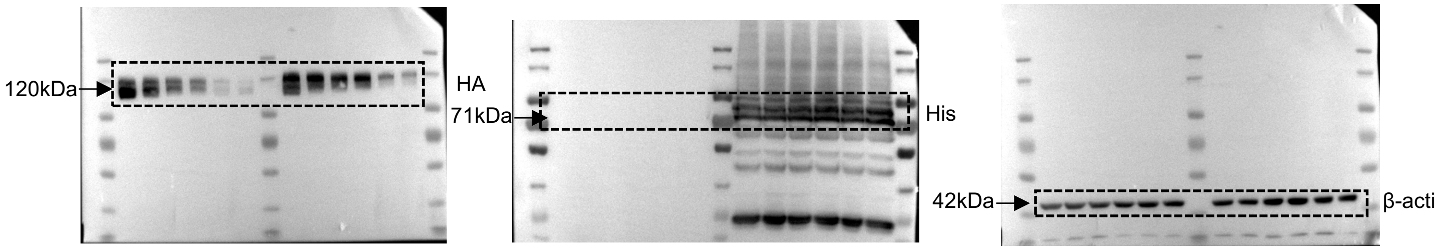

Figure 3F

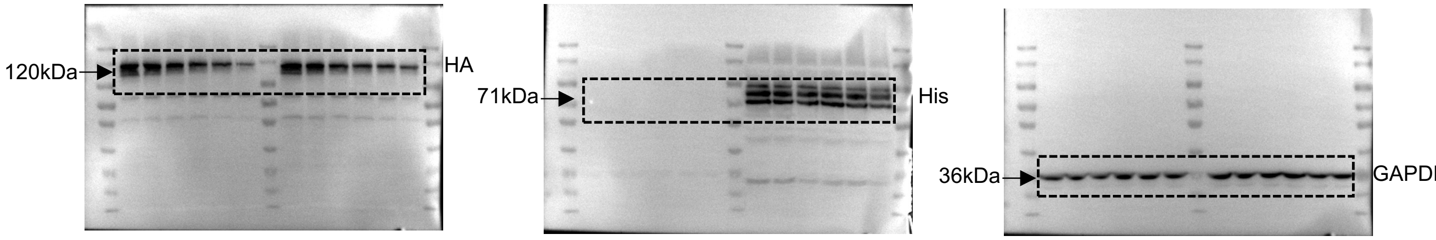

Figure 4A U251 IP

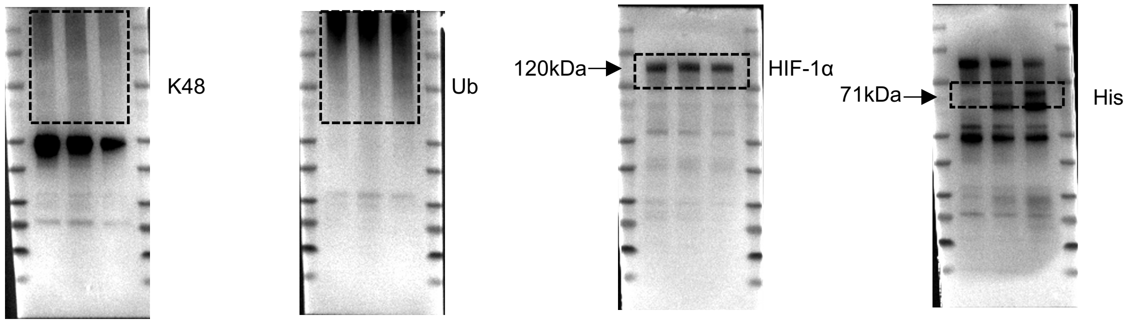

Figure 4A U251 input

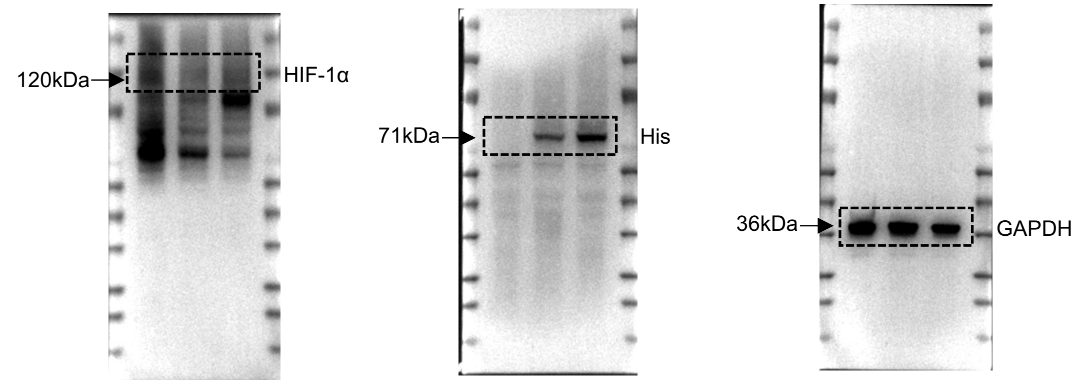

Figure 4A U118 IP

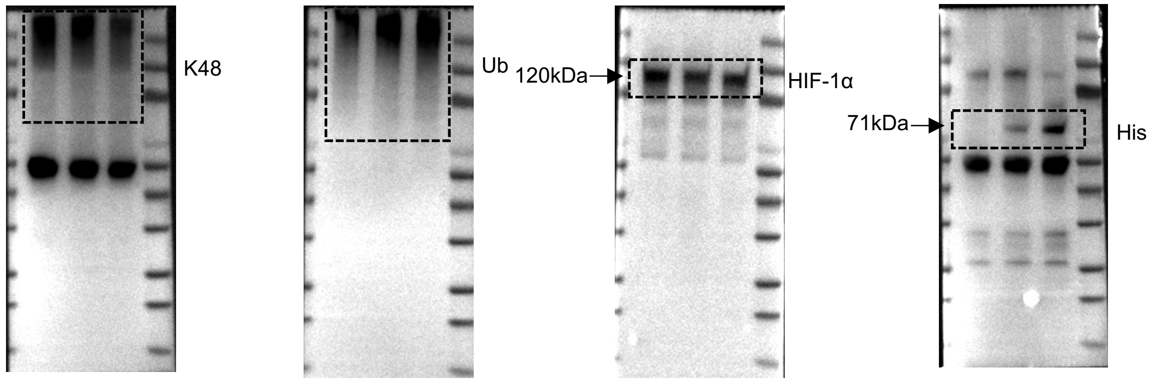

Figure 4A U118 input

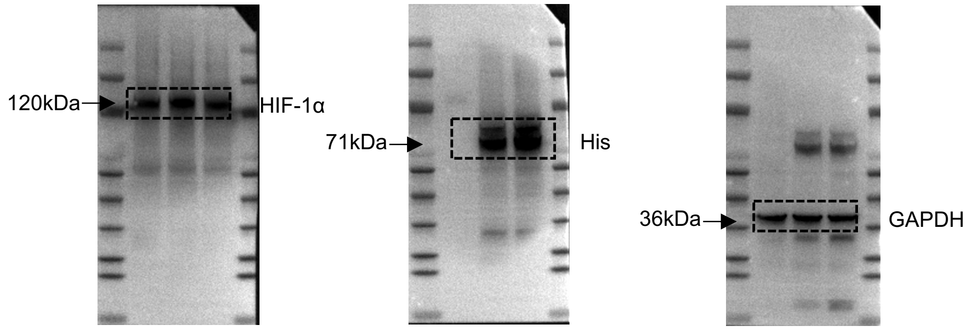

Figure 4B IP

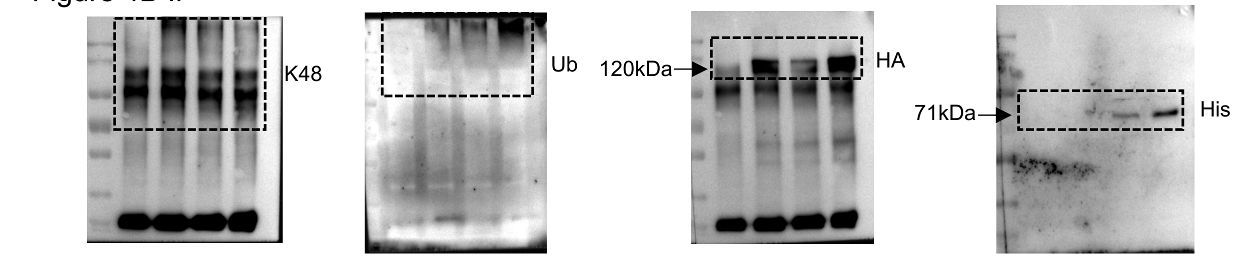

Figure 4B input

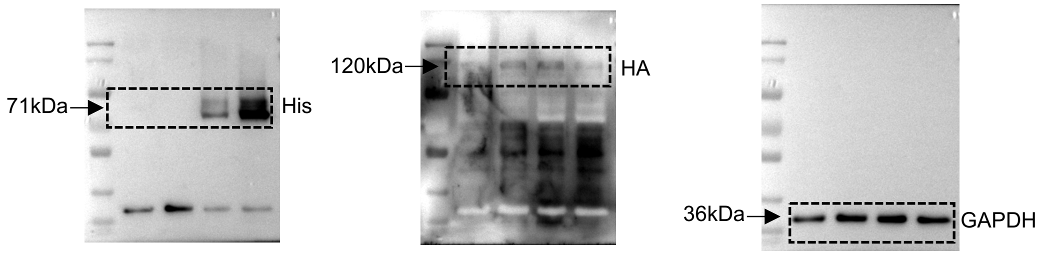

Figure 4C IP

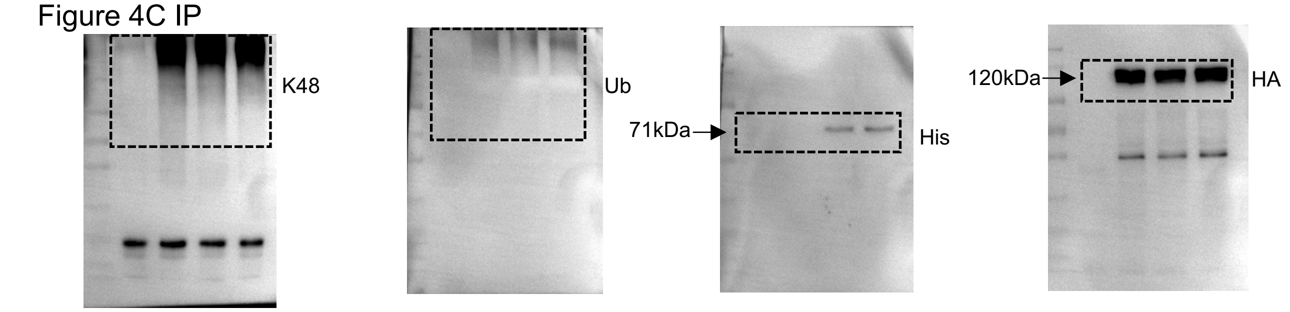

Figure 4C input

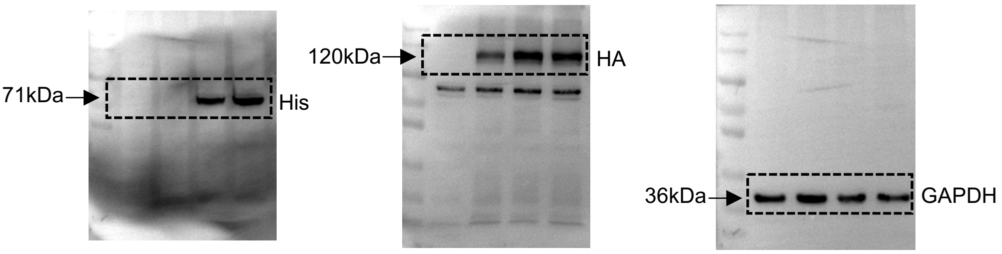

Figure 4D IP

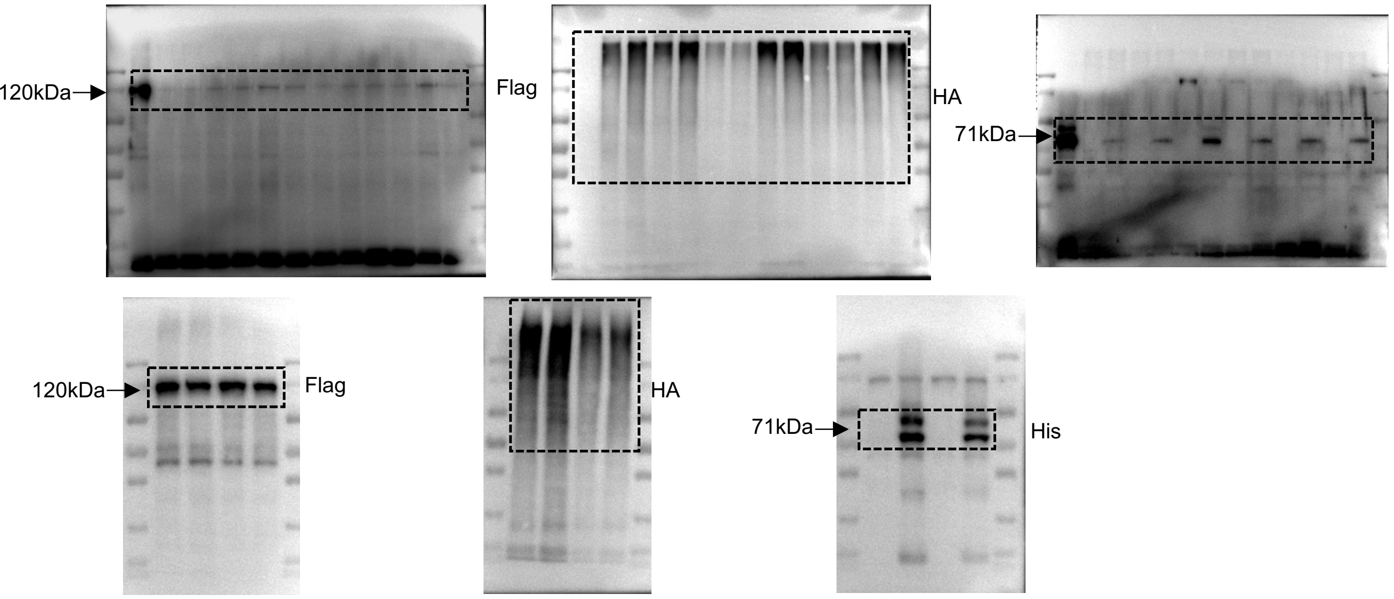

Figure 4D input

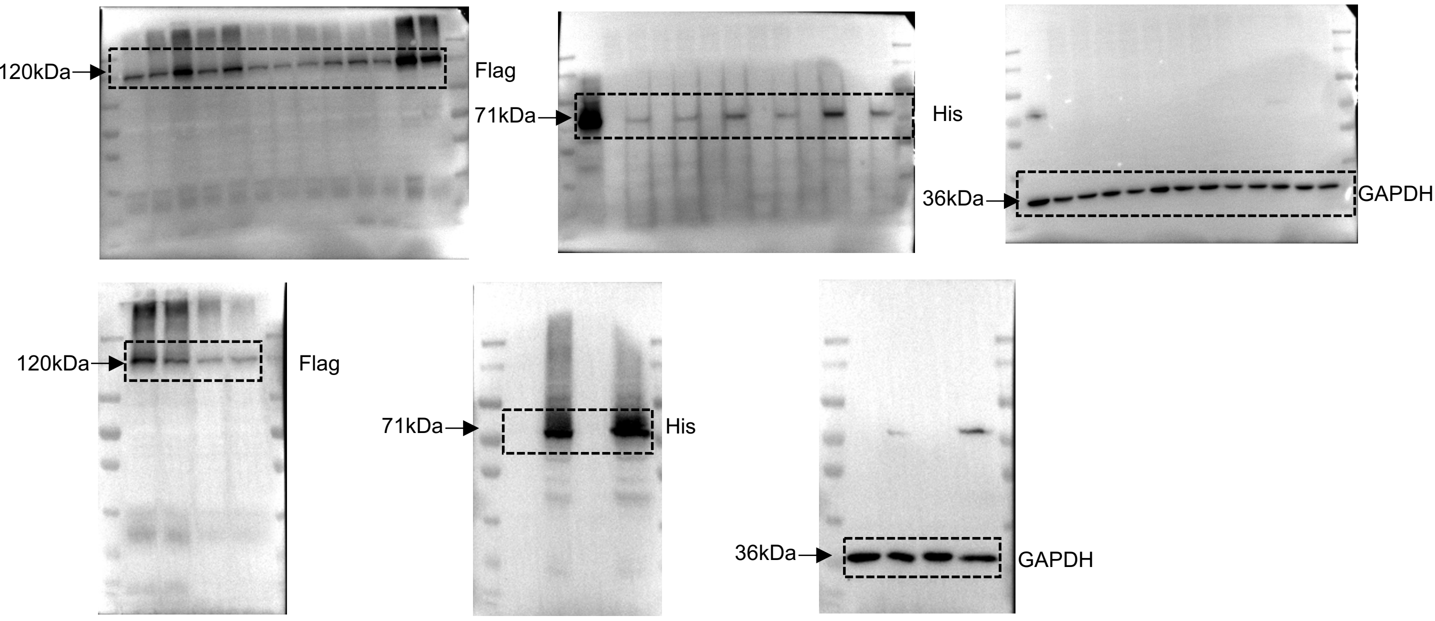

Figure 4E IP

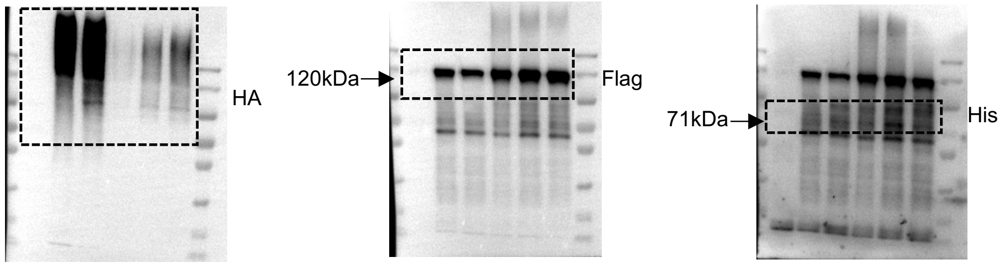

Figure 4E input

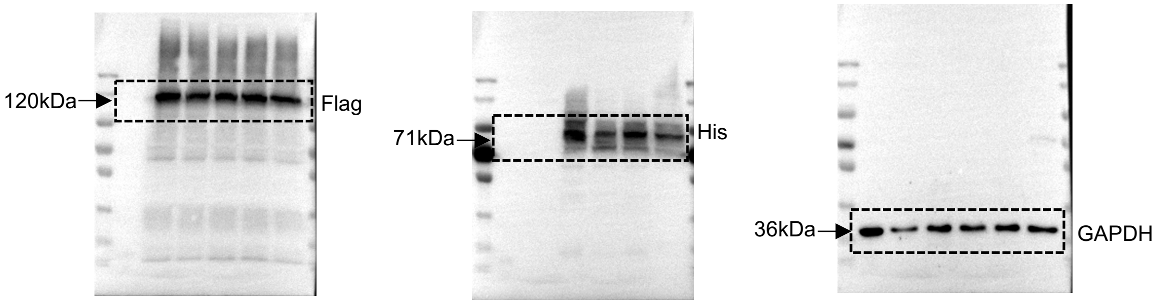

Figure 4F

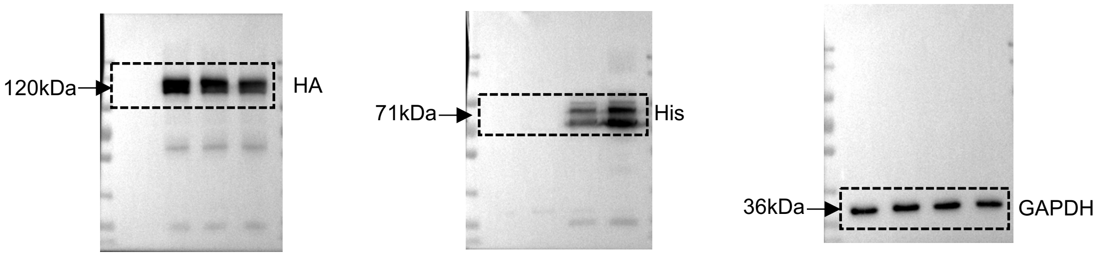

Figure 4G

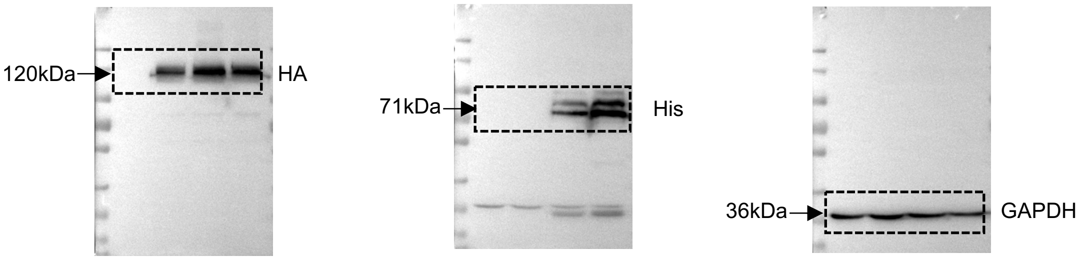

Figure 4H

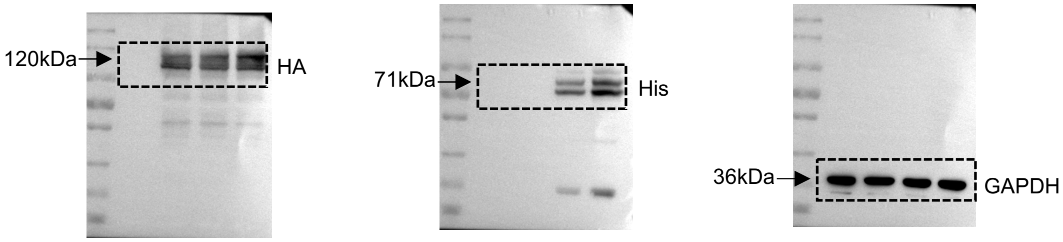

Figure 4I

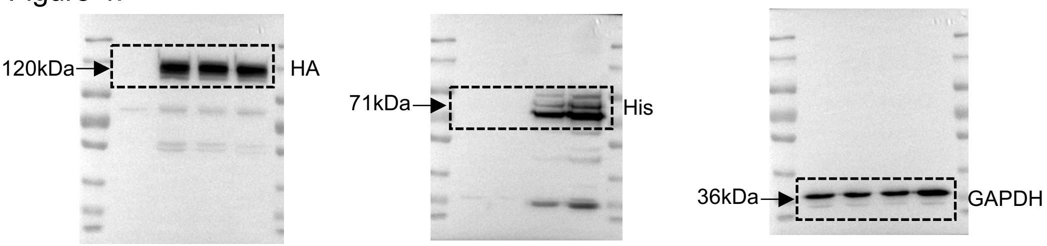

Figure 4J IP

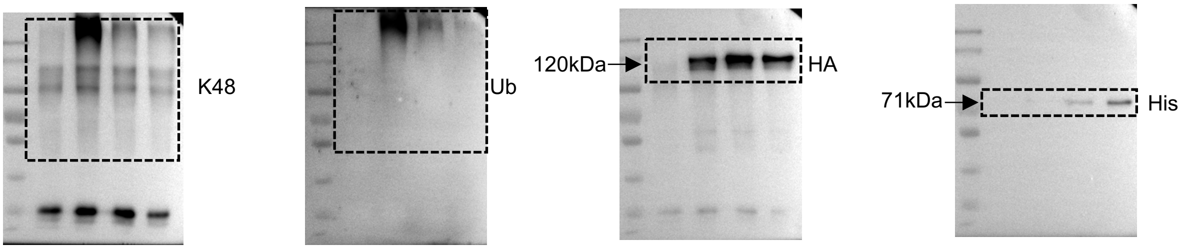

Figure 4J input

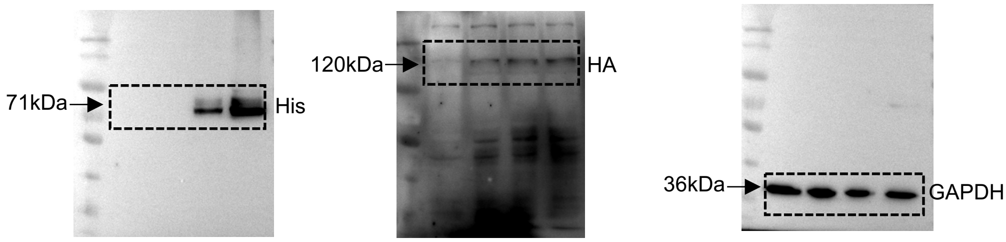

Figure 4K IP

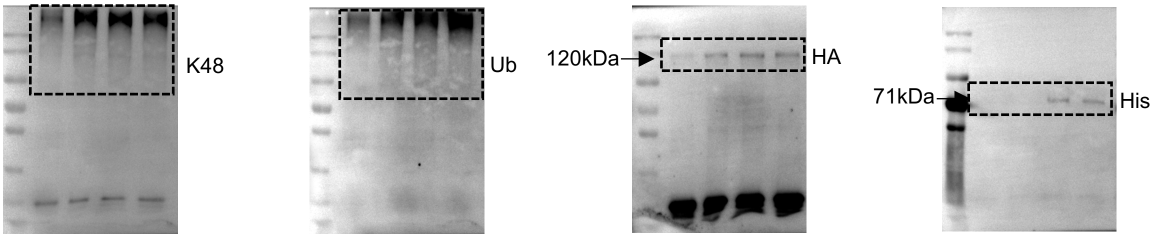

Figure 4K input

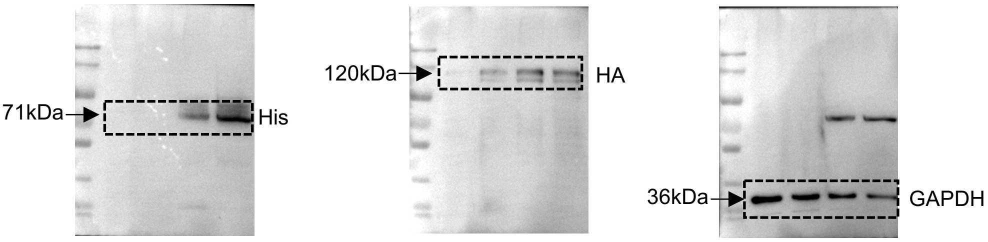

Figure 4L IP

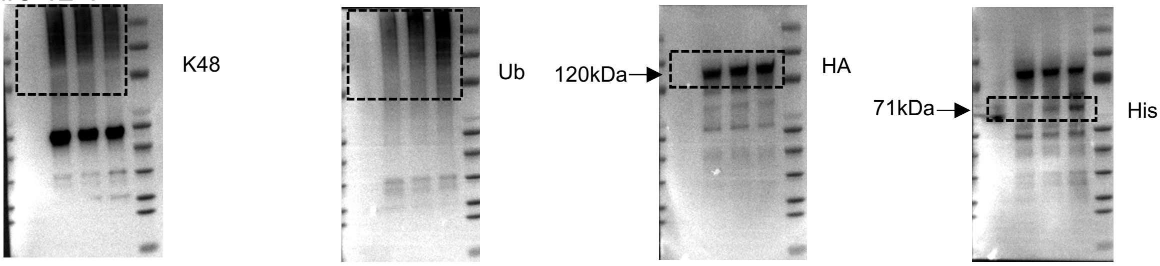

Figure 4L input

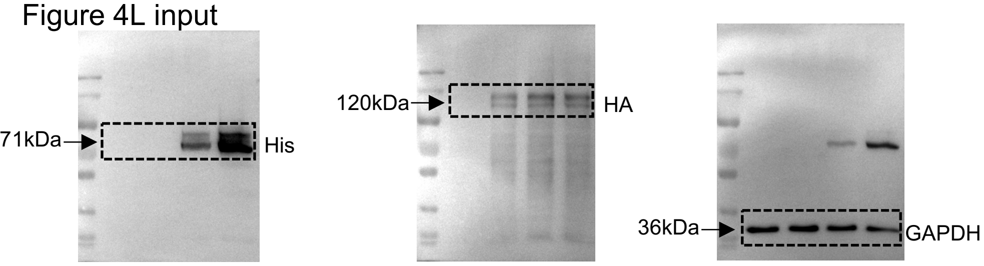

Figure 4M IP

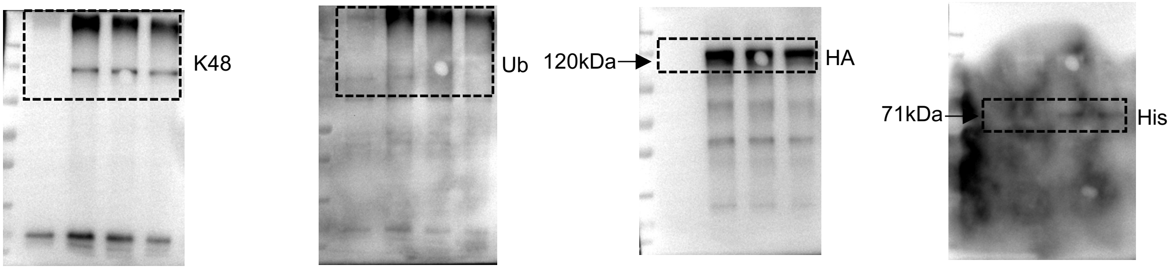

Figure 4M input

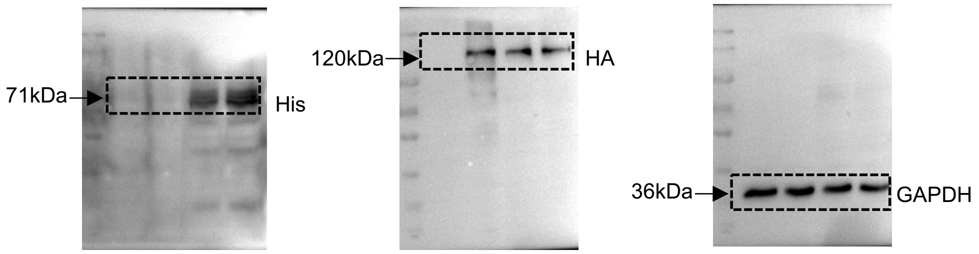

Figure 5A U118

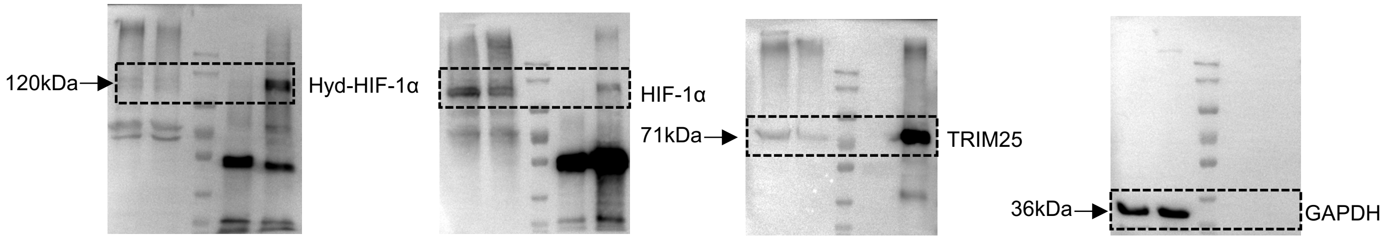

Figure 5A U251

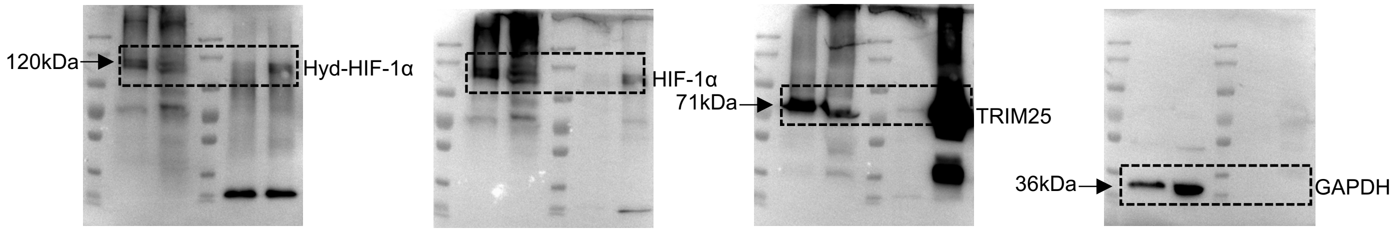

Figure 5B U118

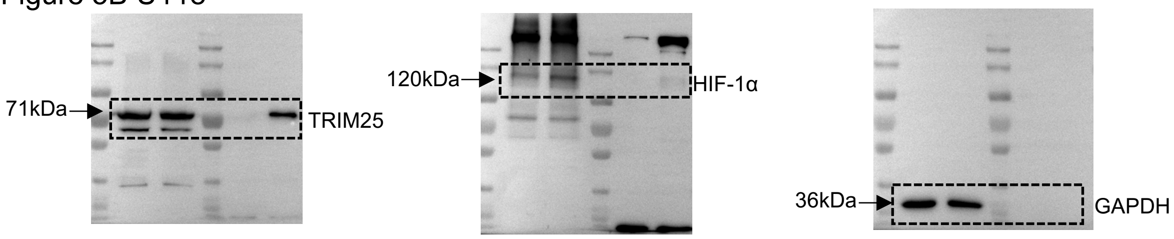

Figure 5B U251

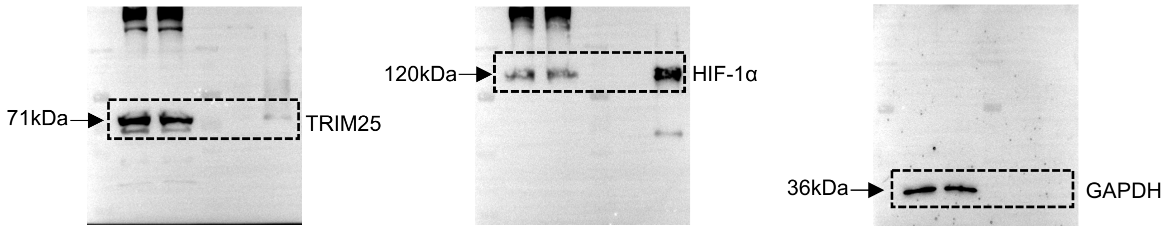

Figure 5C U118

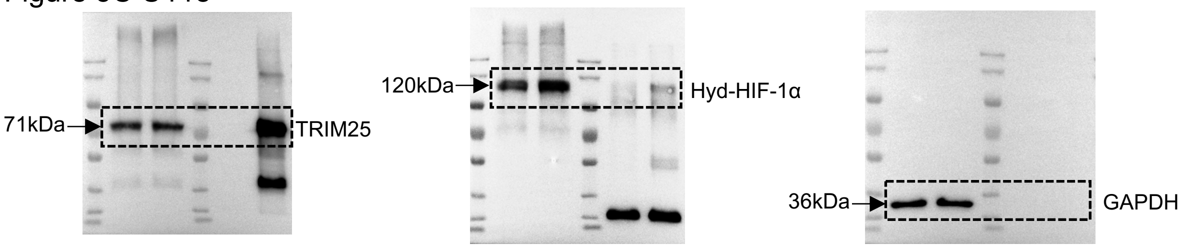

Figure 5C U251

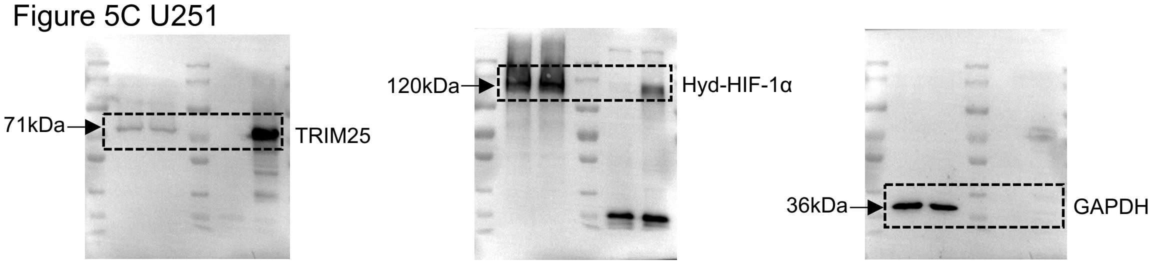

Figure 5D

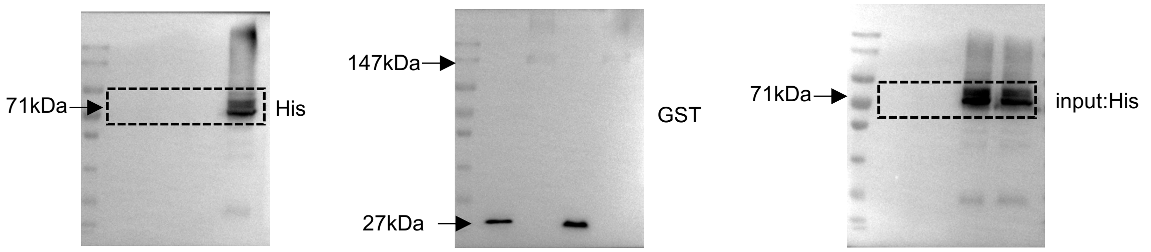

Figure 5E

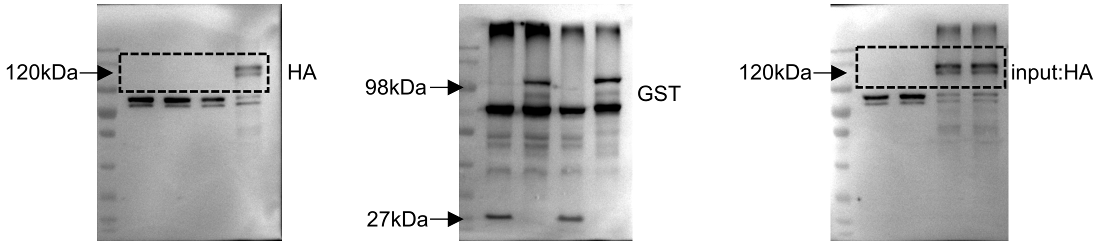

Figure 5F

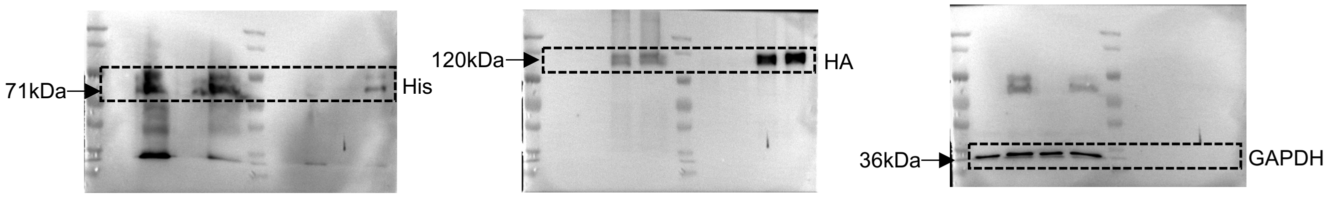

Figure 5G

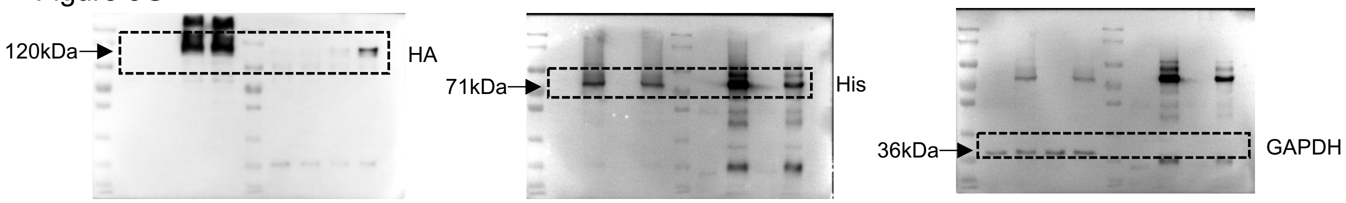

Figure 5H

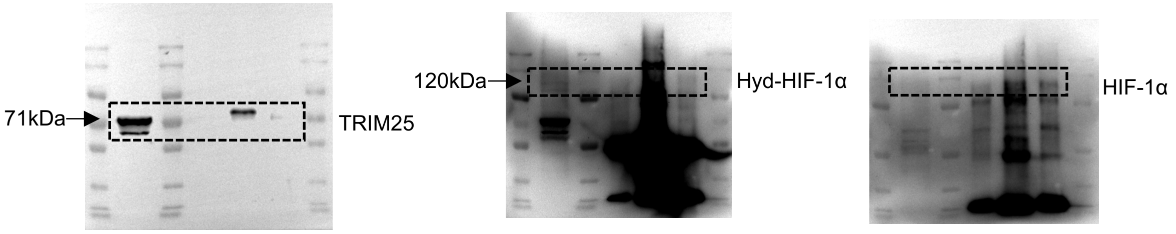

Figure 5I

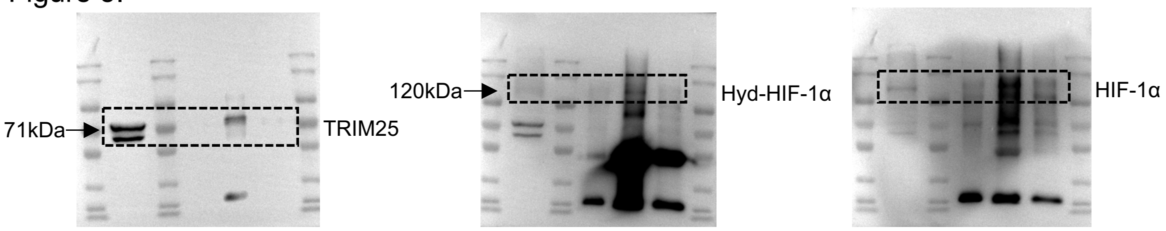

Figure 5J IP

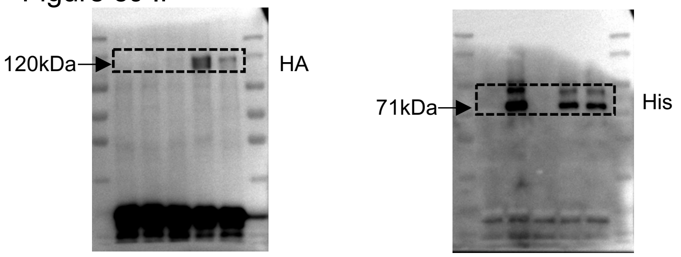

Figure 5J input

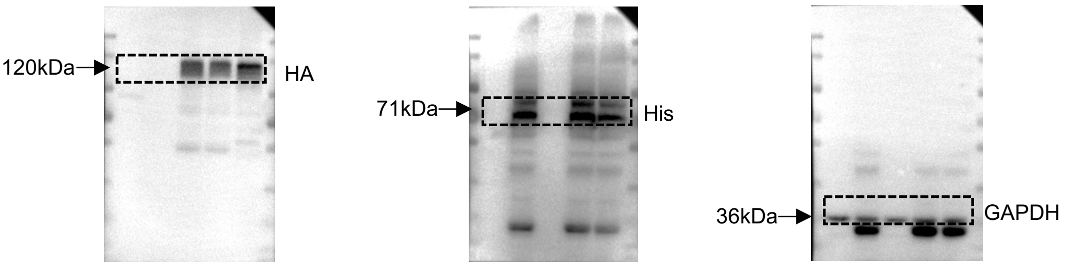

Figure 5K

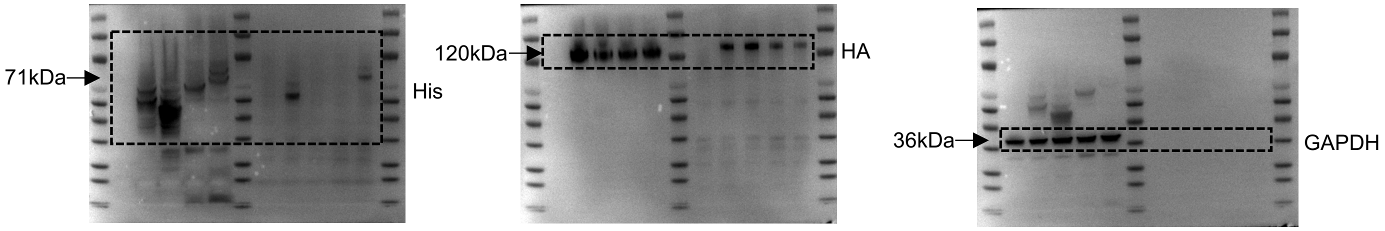

Figure 5L

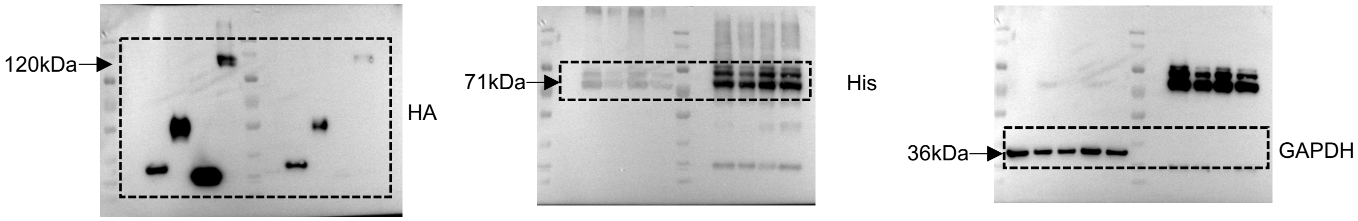

Figure 6A U251

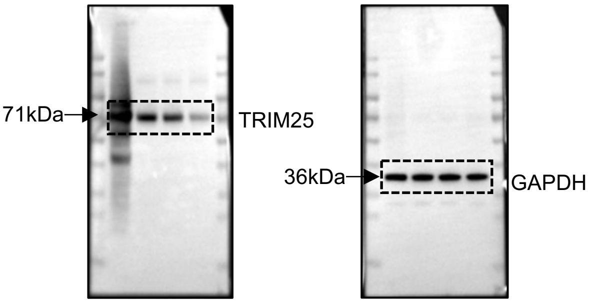

Figure 6A U118

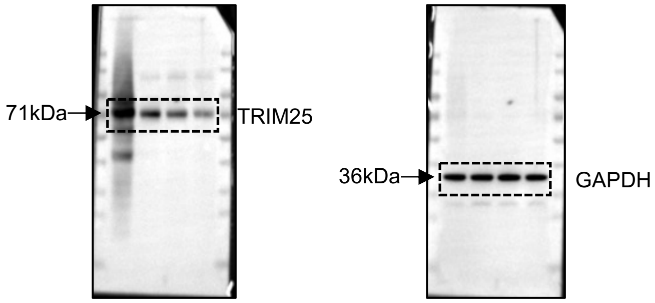

Figure 7F

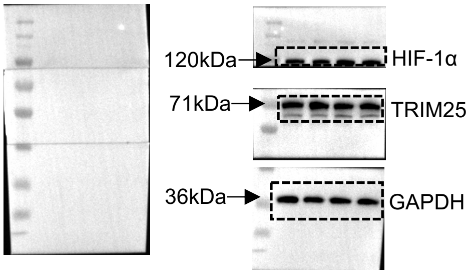

Figure 7G U251

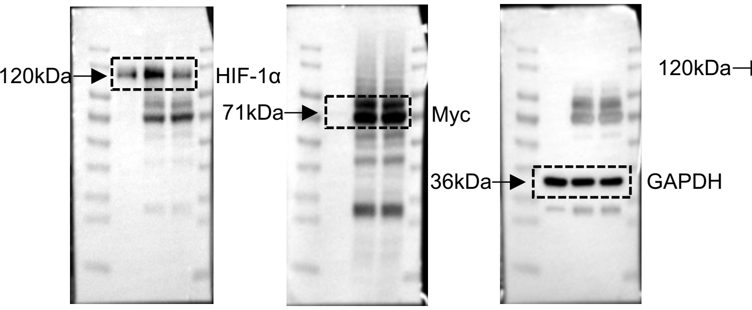

Figure 7G U118

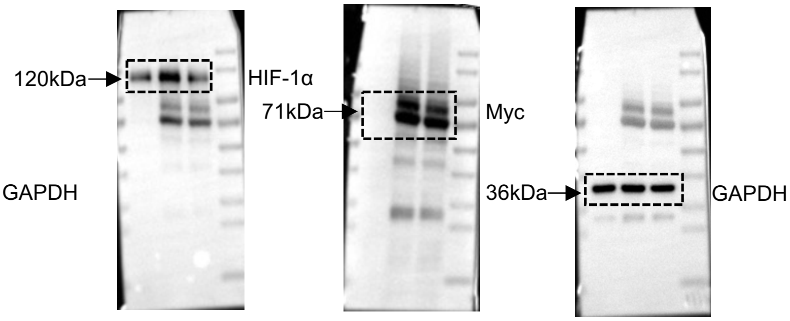

Figure 7H U251

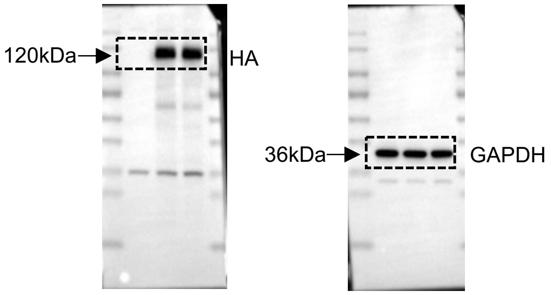

Figure 7H U118

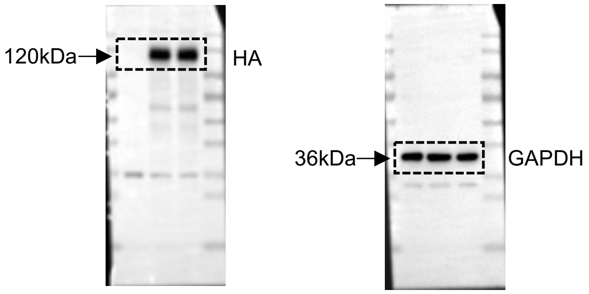

Figure 7I U118

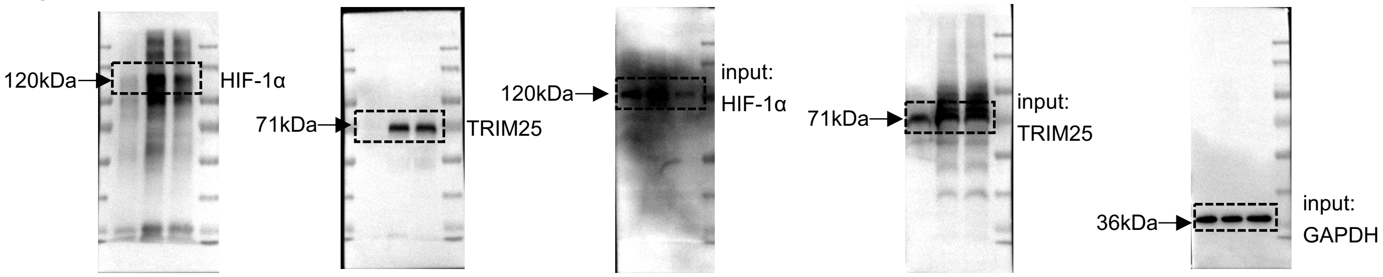

Figure 7I U251

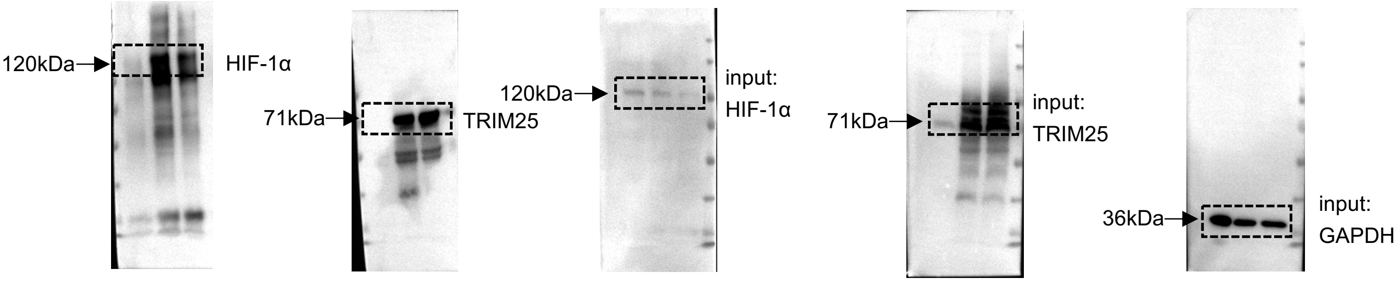

Figure 7J

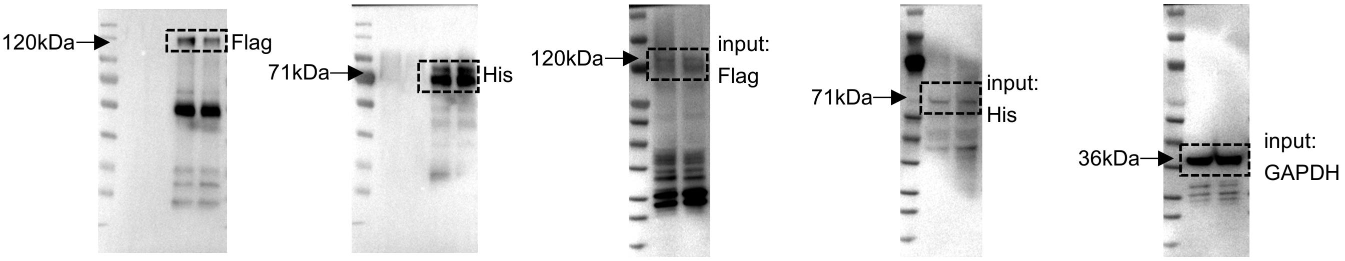

Figure 7K

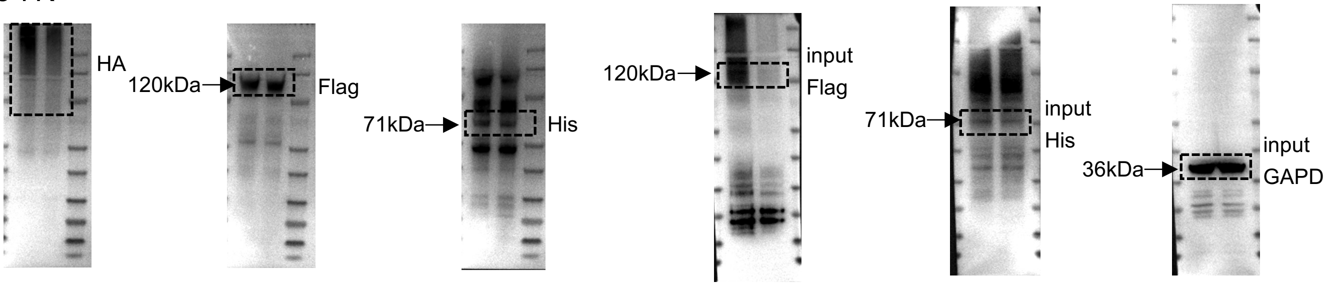

Figure 7L

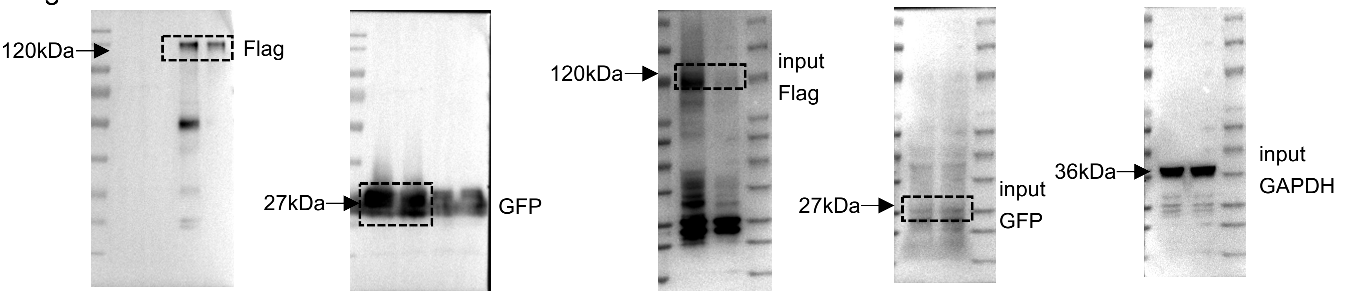

Supplementary Figure

Supplementary Figure 1

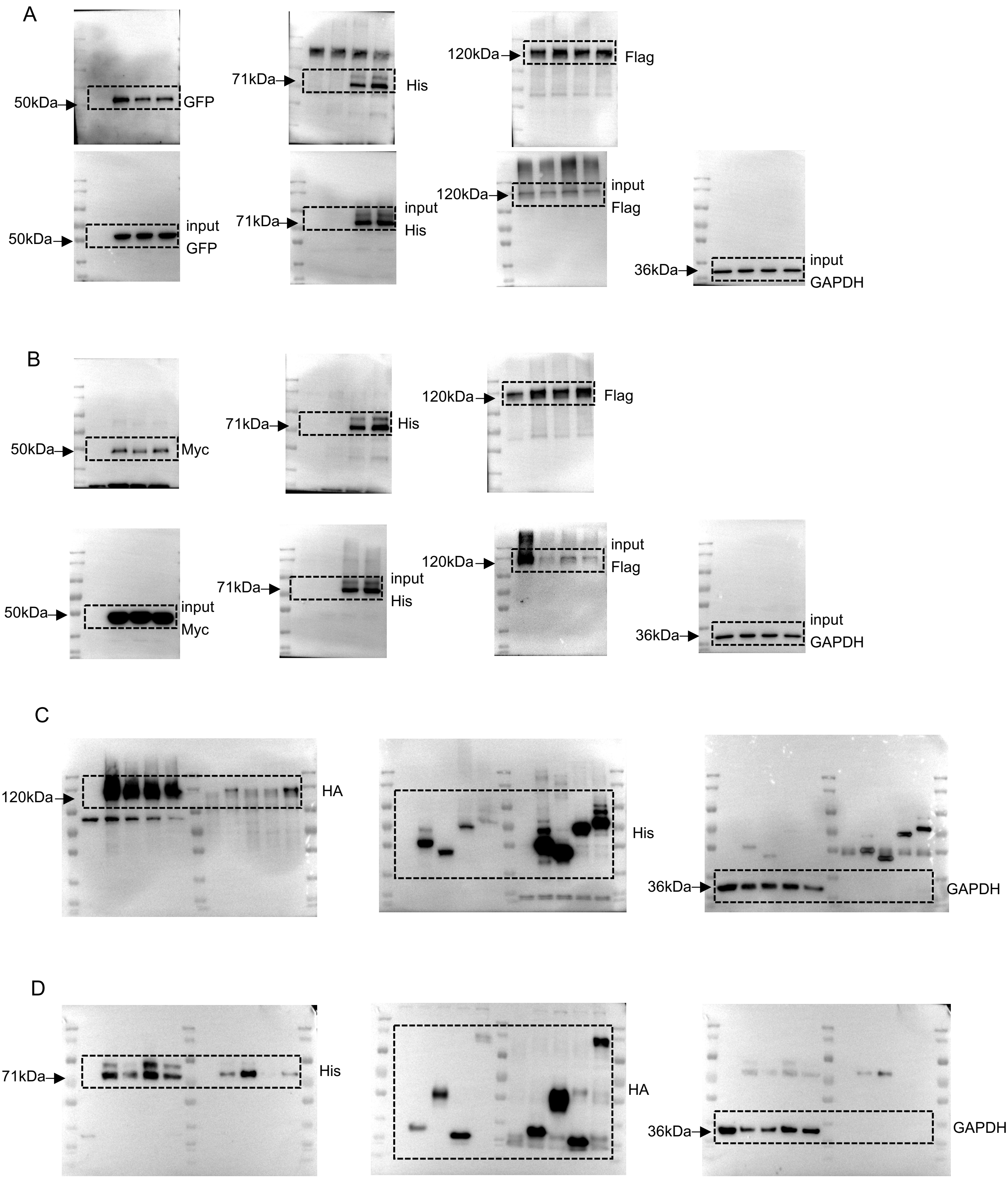

Supplementary Figure 2H

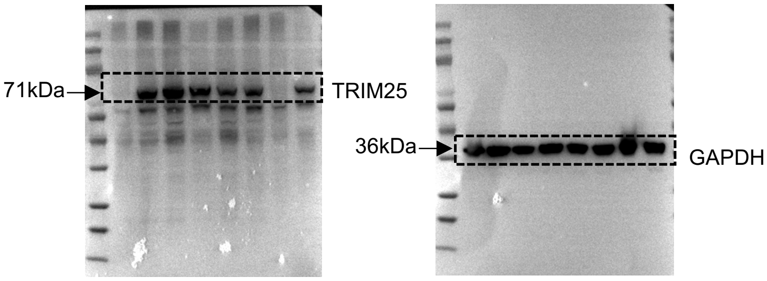

Supplementary Figure 3A U251

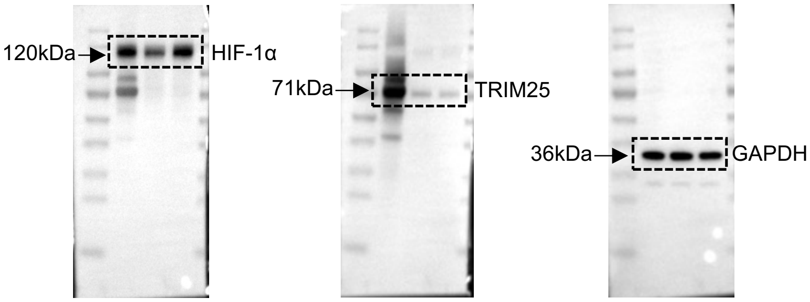

Supplementary Figure 3A U118

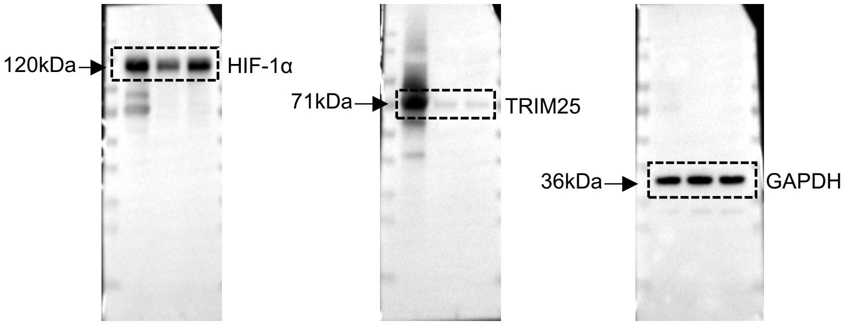

Supplement: Supplementary file 5 — Original Data [file 41419_2026_8757_MOESM5_ESM.pdf]
